# Are electronic nicotine delivery systems helping cigarette smokers quit? Evidence from a prospective cohort study of U.S. adult smokers, 2015–2016

**DOI:** 10.1371/journal.pone.0198047

**Published:** 2018-07-09

**Authors:** Scott R. Weaver, Jidong Huang, Terry F. Pechacek, John Wesley Heath, David L. Ashley, Michael P. Eriksen

**Affiliations:** 1 Division of Epidemiology & Biostatistics, School of Public Health, Georgia State University, Atlanta, GA, United States of America; 2 Tobacco Center of Regulatory Science (TCORS), School of Public Health, Georgia State University, Atlanta, GA, United States of America; 3 Division of Health Management & Policy, School of Public Health, Georgia State University, Atlanta, GA, United States of America; 4 Division of Environmental Health, School of Public Health, Georgia State University, Atlanta, GA, United States of America; Legacy, Schroeder Institute for Tobacco Research and Policy Studies, UNITED STATES

## Abstract

**Background:**

The potential of electronic nicotine delivery systems (ENDS) to reduce the cardiovascular and other disease risks of smoking is of great interest. While many smokers report using ENDS for cessation, their impact under real-world use patterns and conditions on adult smokers’ quitting behavior is uncertain. The objective of this study was to generate more recent and comprehensive evidence on the effect of “real world” ENDS use on the population quit rates of adult smokers while taking account of frequency and duration of use, device type, e-liquid flavor, and reasons for use.

**Methods and findings:**

We conducted a population-based, prospective cohort study of a random probability sample of 1284 U.S. adult smokers recruited in August/September 2015 and re-contacted one-year later (September 2016) from GfK’s KnowledgePanel, a national, probability-based web-panel designed to be representative of non-institutionalized U.S. adults. Among the 1081 baseline smokers who remained members of KnowledgePanel, 858 completed the follow-up survey. The primary outcome was smoking abstinence for at least 30 days prior to follow-up. Secondary outcomes were making a quit attempt during the 12-month study period and number of cigarettes smoked per day at follow-up. The adjusted odds of quitting smoking were lower for those that used ENDS at baseline (9.4%, 95% CI = 5.22%-16.38%; AOR = 0.30, 95% CI = 0.13–0.72) compared to smokers who did not use at ENDS (18.9%, 95% CI = 14.24%-24.68%). Smokers who used ENDS daily at some point during the study period were also less likely to quit smoking than nonusers (AOR = 0.17; 95% CI = 0.04–0.82). Limited ability to draw causal inferences from the observational design and a lack of biochemical verification of quitting smoking or ENDS use are limitations of this study.

**Conclusions:**

We found no evidence that ENDS use, within context of the 2015–2016 US regulatory and tobacco/vaping market landscape, helped adult smokers quit at rates higher than smokers who did not use these products. Absent any meaningful changes, ENDS use among adult smokers is unlikely to be a sufficient solution to obtaining a meaningful increase in population quit rates. Additional research is needed to reconcile the divergent literature and monitor the impact of ENDS in an environment of rapidly evolving markets and regulatory policies.

## Introduction

Electronic nicotine delivery systems (ENDS) have generated significant interest for their potential cardiovascular effects, as well as their potential to reduce the cardiovascular disease and other health risks of smoking [1–8]. Whereas several tobacco control experts have expressed support for the harm reduction potential of ENDS [9–20], a spirited debate has ensued [21–25]. Two of the central pillars on which the harm reduction argument rests are: (a) use of ENDS is substantially less harmful than smoking cigarettes and (b) their use leads to higher population-level smoking quit rates [6,13,26–30]. Although more research is necessary before the full extent of the risks from ENDS use are known [31,32], the extant research suggests that use of ENDS is likely substantially less harmful than smoking combustible cigarettes [25,33–37], with most debate focusing on how much less harmful [28]. Reduced risk, however, is insufficient for achieving population-level harm reduction without effecting switching from a higher risk to a lower risk product. Nearly one-half of smokers reported having ever tried and approximately one in six currently using ENDS in 2014 [38], with more recent data suggesting these numbers increased in 2015 [39]. Quitting and/or reducing the harms of smoking are cited as the main reasons smokers use ENDS [40], and some smokers have credited ENDS with helping them to successfully quit smoking [41,42]. Two randomized controlled trials (RCTs), conducted in New Zealand and Italy, found limited support for their efficacy in smoking cessation [43,44]. Whereas the effects of ENDS with nicotine compared to placebo (non-nicotine) ENDS were non-significant in these two studies, the pooled effect was statistically significant [45]. One of these studies also found higher cessation, though not statistically significant, for ENDS compared to the nicotine patch; however, participants’ access to products differed in the two conditions [44]. A recent naturalistic RCT found that, whereas ENDS were associated with reduced smoking, the numerically positive effect on quit attempts and abstinence was not significant [46]. In contrast, two meta-analyses of primarily longitudinal cohort studies found lower odds of quitting among ENDS users, casting doubt on the claim that observed declines in U.S. adult population smoking rates can be attributed to ENDS [47,48]. Reflecting the conflicted and nuanced scientific literature, two other meta-analytic studies with different study-eligibility criteria found either no significant effect [49] or a positive effect of ENDS use on quitting [50]. However, the quality of extant evidence has been rated very low in several reviews [45,48,49,51], prompting the US Preventive Services Task Force to conclude the evidence insufficient to recommend ENDS for smoking cessation [52]. More recently, a National Academies of Science committee concluded that there “is limited evidence that e-cigarettes may be effective aids to promote smoking cessation,” but “there is moderate evidence from observation studies that more frequent use of e-cigarettes is associated with an increased likelihood of cessation” [37].

Well-controlled RCTs can provide critical evidence of the potential of ENDS for effecting smoking cessation, whereas well-designed longitudinal cohort studies can offer unique and important insights on the population-level effectiveness of ENDS under “real world” use and conditions [37,47,53,54]. However, insights gleaned from past longitudinal cohort studies have been clouded by methodological limitations [21,48,51,53], specifically insufficient attention to the motivations and intentions for using ENDS, characteristics of the ENDS device related to the delivery of nicotine, overall user satisfaction, and frequency of use [37,53]. Smokers note many different reasons for using ENDS [55,56], and accounting for whether they use ENDS primarily to quit smoking or for other reasons (e.g., to use in situations where smoking is not permitted) is important [56,57]. Even when the primary reason for using ENDS is to quit smoking, certain device types and patterns of use may be more conducive to quitting than others [36,53,58]. ENDS with nicotine delivery profiles comparable to the combusted cigarette may better alleviate smokers’ nicotine cravings and serve as a more acceptable alternative for cigarettes. In contrast to disposables and many rechargeable/cartridge-based ENDS, newer and later generation ENDS that are predominantly open-systems with more powerful batteries have produced nicotine delivery profiles more comparable to combusted cigarettes [59,60]. However, the few studies that have examined the effect of device type on quitting smoking have yielded mixed results [56,61]. Smoking behavior may also be affected by e-liquid flavor, though this research remains limited [62–65]. To our knowledge, no RCTs and only one prospective cohort studies of ENDS and smoking have considered the effect of e-liquid flavor despite important implications for regulatory policy [66]. Results from multiple cross-sectional survey studies using large, nationally representative samples have provided compelling evidence that frequent use (daily or at least >5 times in the last month) is associated with recent former smoker status [67–69]. Two cohort studies have found limited evidence in support of a positive association between daily ENDS use with subsequent substantial reduction in cigarettes smoked, cessation attempts, and, for one of these studies, increased quitting if using a tank system, compared to non-users [61,70]. However, other research has found either no effect of frequency (or device type) on smoking abstinence [56] or an association of more frequent ENDS use with subsequent greater quantity and frequency of smoking [71]. Another study found no difference in smoking abstinence at follow-up between daily ENDS users and non-users; whereas non-daily users were less likely to be abstinent compared to non-users [72].

In summary, the research on the impact of ENDS use on adult smokers’ quitting remains inconsistent and methodologically limited. Further, due to the rapid evolution in technology and marketing of ENDS, along with population shifts in patterns of use [36,57], results of older studies may not apply to the present [37]. Therefore, the objective of this national, prospective cohort study is to generate more recent and comprehensive evidence on the effect of “real world” ENDS use on the population quit rates of adult smokers while addressing key limitations of prior studies, specifically by taking account of frequency of use, device type, e-liquid flavor, and reasons for use. We hypothesized that ENDS use among smokers would be prospectively associated with quitting outcomes after adjusting for baseline differences in potential confounding factors, and this association would depend on device, use patterns, and intensions for use.

## Methods

### Study design and participants

Participants were recruited from GfK’s KnowledgePanel, a national, probability-based web-panel designed to be representative of non-institutionalized U.S. adults. For this prospective cohort study, a sample of 1284 current, established smokers at baseline was identified among respondents to the 2015 (August-September) Tobacco Products and Risk Perceptions Survey (TPRPS) for a 12-month follow-up study on their smoking and ENDS use. A study completion rate of 76.0% was obtained for the baseline survey. In August-October 2016, 1018 baseline current smokers who had remained members of GfK KnowledgePanel were invited to complete the follow-up survey, which yielded 858 respondents (66.8% of the baseline smokers; 84% of those invited for the follow-up survey). The institutional review board of the Georgia State University approved this study with a waiver of informed consent.

### Outcome variables

The primary outcome variable was smoking abstinence for at least 30 days at follow-up measured by responding (a) “not at all” to “Do you now smoke cigarettes every day, some days, or not at all?” and (b) “no” to “In the past 30 days, have you smoked a cigarette, even one or two puffs?” Secondary outcome variables were (a) making at least one attempt to quit smoking completely since the baseline survey, including successful quit attempts, and (b) among those smoking at follow-up, the average number of cigarettes smoked per day (CPD). Detailed information about these measures can be found in S1 Table.

### Primary ENDS exposure variables

All survey participants were shown preamble text with pictures describing ENDS and their different features. When answering questions about their ENDS use, participants were instructed to “think only about use of these products without marijuana, marijuana concentrates, marijuana waxes, THC, or hash oils.” Current ENDS use at baseline was then assessed by “Do you now use electronic vapor products every day, some days, rarely, or not at all?” Smokers who reported using ENDS “every day,” “some days,” or “rarely” were defined as baseline ENDS users (n = 248), whereas those reporting “not at all,” or, on prior questions never use or no awareness of ENDS were defined as baseline nonusers (n = 606). In addition, we separately classified smokers by whether they used ENDS during the study period spanning from baseline survey to follow-up. Participants who reported current use of ENDS at baseline and/or follow-up, any past 30-day use of ENDS at follow-up, or any use of ENDS since the baseline survey were classified as any ENDS users (n = 347), whereas those who reported no current use at baseline and at follow-up and no use in between baseline and follow-up were classified as nonusers during the study period (n = 507). Those who reported any ENDS use during the 12-month study period were further subdivided as follows: (a) ENDS use at both baseline and follow-up (n = 129), (b) ENDS use during the study period but not at baseline (n = 53), or (c) ENDS use at or after baseline but not at follow-up (n = 165). Frequency of ENDS use, importance of quitting smoking as reason for using ENDS, and ENDS product characteristics (flavor and device type) were assessed as potential effect modifiers of ENDS use on quitting smoking. We operationalized each as follows (see S1 Table for more details regarding their measurement):

1. Smokers who used ENDS were classified as daily ENDS users if they reported daily use of ENDS or using ≥25 days during the past 30 days at either baseline or follow-up (n = 53).
2. To assess whether smokers were using ENDS for quitting or for other reasons, they were asked to indicate how important ENDS were to help them “quit smoking regular cigarettes” on a 7-point scale (0 = Not at all important to 6 = Very important). Quitting smoking was considered an important reason for using ENDS if a smoker responded 3 or higher (0 = not at all important to 6 = very important) (n = 248).
3. At both baseline and follow-up, ENDS users were asked to indicate among a list of 10 flavor categories, including “tobacco flavor,” which flavors they usually used (or “last used” if they were no longer using ENDS at follow-up). They were coded as (a) tobacco flavor or unflavored user if they selected only tobacco flavor or unflavored at baseline and follow-up (n = 96); (b) a menthol/wintergreen/mint flavor user if they indicated they selected this flavor at baseline or follow-up, but no other flavor other than tobacco flavor or unflavored (n = 57); (c) other flavor user if they selected any flavor other than tobacco or menthol/wintergreen/mint at baseline or follow-up (n = 174).
4. ENDS users were asked at baseline and follow-up if the device they used most of the time was (a) rechargeable, (b) used cartridges (if rechargeable), or (c) used a tank system (if rechargeable but did not use cartridges). If they reported using a tank system, they were classified as a tank user (n = 87); if they reported using a cartridge system but no tank system, they were classified as a cartridge user (n = 113); else if were coded as a disposable/other ENDS user (n = 48).

### Adjustment variables

Sociodemographic variables, smoking history and intensity, quit intentions and history, other combustible tobacco use, physical health, prior mental health treatment, and alcohol use were identified as potential confounders and measured at baseline. Smoking dependence was measured separately by (a) intensity of smoking (i.e., average number of cigarettes per day), (b) perceived addiction to smoking, and (c) strength of cravings to smoke cigarettes. Length of smoking was measured by number of years smoked. Motivation to quit smoking was measured separately by (a) reported intentions to quit smoking, (b) number of past-year quit attempts, (c) prior use of FDA-approved pharmacological treatments for smoking cessation, and (d) regret having started smoking. Dual/poly combustible tobacco use was measured by items assessing concurrent use of traditional cigars, little cigars and cigarillos, or hookah. Other respondent characteristics were measured by questions from profile surveys pre-administered by GfK to all KnowledgePanel members assessing: (a) physical health (self-reported physical health status and whether they have been diagnosed with asthma, chronic bronchitis or COPD); (b) prior mental health treatment (having ever seen a psychiatrist, psychologist, or social worker for counseling or therapy); (c) past month consumption of alcohol; and (d) sociodemographic characteristics (e.g., age, gender, race/ethnicity, sexual orientation, education, income). To address potential panel conditioning bias, the number of smoking-related studies completed by the respondent in the past year was computed by GfK and controlled for in the analysis. Detailed information about these measures can be found in S1 Table.

### Measures of methods used to quit smoking

In order to better interpret results of primary results regressing smoking outcomes on ENDS exposure variables, we also assessed the methods and resources smokers used in their attempts, either successful or unsuccessful, to quit smoking. If a participant reported they had completely quit smoking for good, they were asked “When you quit smoking for good, did you do any of the following?”: responding *yes*/*no* to (a) “gave up cigarettes all at once?” (cold turkey); (b) “gradually cut back on cigarettes?”; (c) “switched completely to electronic vapor products, such as…?”; (d) “substituted some of my regular cigarettes with electronic vapor products, such as…?”; (e) “used nicotine replacements like the nicotine patch, nicotine gum, nicotine lozenges, nicotine nasal spray, or nicotine inhaler?”; (f) “used medications like Wellbutrin, Zyban, buproprion, Chantix, or varenicline?”; (g) got counseling, help from a telephone help or quit line, a website such as Smokefree.gov, books, pamphlets, videos, a quit tobacco clinic, class, or support group, or an internet or web-based program, or from a doctor or other health professional?”; (h) “used little cigars, filtered cigars or cigarillos to quit smoking cigarettes?”; (i) “used any of the following: traditional cigars, snus, chewing tobacco, dip or snuff, dissolvables, hookah, or ‘heat-not-burn’ to quit smoking cigarettes?; and (j) “relied on the support of friends and family to help you quit smoking cigarettes?” If the participant was still smoking at the follow-up survey, they were asked to report the methods or resources they had used to try to quit smoking since the baseline survey. Detailed information about these measures can be found in S1 Table.

### Statistical analysis

We first calculated proportions and their 95% confidence intervals for ENDS use at baseline and for smoking and ENDS use at follow-up among baseline dual users. We then used weighted logistic regression or weighted general linear models to assess whether ENDS users were more likely to be smoke daily at baseline and whether they differed on study covariates. For our primary analyses, associations between ENDS use and binary outcomes (i.e., making a smoking quit attempt and 30-day smoking abstinence), controlling for potential confounders, were estimated by adjusted odds ratios and 95% confidence intervals from weighted logistic regression models. Weighted general linear models were used to estimate the association between ENDS use and CPD among those participants still smoking at follow-up, controlling for potential confounders. For all primary analyses, models were estimated separately for each operational definition of ENDS exposure (viz., baseline ENDS use vs. nonuse at baseline; any ENDS use and sub-patterns of any ENDS use vs. no use during the study; by frequency of ENDS use; by importance of ENDS use for quitting; by flavor use; and by device type). When the ENDS exposure variable had more than two levels, exploratory pairwise tests were conducted. Furthermore, all primary analyses were repeated while restricting the sample to participants who were daily smokers at baseline. Finally, among smokers who reported a quit attempt during the study, either successful or unsuccessful, we estimated weighted proportions and associated 95% confidence intervals for each assessed method or resource used during their quit attempt(s), stratified by their use of ENDS and whether they were 30-day abstinent from smoking at the follow-up survey.

For all analyses, a study-specific post-stratification weight, based on demographic and geographic benchmarks from the March 2015 Current Population Survey, was used to adjust all analyses for sources of sampling and non-sampling error. Missing data were handled using two different approaches. The first approach involved a complete-case analysis whereby participants missing data on one or more variables in a model were excluded from that analysis. A post-stratification weight variable that adjusts for attrition bias was used with this approach. For the second approach, we used the Mplus statistical package (v. 8) to generate 50 imputed datasets based on Bayesian Monte Carlo Markov Chain (MCMC) estimation of an unrestricted mean and variance covariance model, which included all analysis variables and additional variables from the baseline survey that were predictive of missingness. The fraction of missing information ranged .20 to .52 for parameter estimates of key interest to this study. As the general pattern of results were similar between the two approaches, results from the complete-case analysis with weight adjustment for missingness are presented in this paper, and results from the multiple-imputation approach are presented in S2–S6 Tables. The few instances where differences in patterns of statistical significance were observed between the two approaches are noted in text. A two-tailed α = .05 was set *a priori* for all analyses, which were conducted using the Survey package (v. 3.31.5) for the R statistical program (v. 3.4.0) [73,74].

## Results

### Descriptive data

Among smokers who completed the follow-up survey, 27.1% (95% CI: 22.6%, 32.0%) reported using ENDS at baseline. One year later, 90% of dual users were still smoking. Over half (53.5%, 95% CI = 43.5%, 63.1%) continued to smoke and use ENDS, and 37.4% (95% CI = 28.6%, 47.1%) were still smoking but had discontinued ENDS. Only 9.2% (95% CI: 5.1%, 15.8%) reported having quit smoking at follow-up (Table 1).

**Table 1:** Smoking and ENDS use at one year follow-up for baseline dual users (Smoker + ENDS user) (N = 248).

	n	wt. %	95% CI
**Quit Smoking and Quit ENDS**	17	6.67	3.23, 13.24
**Quit Smoking, Using ENDS**	9	2.49	1.02, 5.98
**Current Smoker, Quit ENDS**	102	37.37	28.60, 47.06
**Dual User (Current Smoker and Using ENDS)**	120	53.47	43.56, 63.11

ENDS = electronic nicotine delivery systems. wt. = weighted. CI = confidence interval.

We also examined whether those who used ENDS were more likely than non-users to be daily smokers at baseline. Among smokers who did not use ENDS at baseline, 73.5% (95% CI: 66.8, 81.2%) smoked daily compared 70.5% (95% CI: 60.6, 78.7%) among those who were using ENDS at baseline (p = .56). Similarly, there was no statistically significant difference in the proportion of daily smokers among those who used ENDS at any point during the study and those that did not: 74.7% (95% CI: 66.8, 81.2%) and 71.4% (64.8, 77.2%), respectively (p = .50).

Covariate distributions for those that used ENDS at any point during the study and for non-users are reported in Table 2. Compared to smokers who did not use ENDS, smokers who used ENDS during the 12-month study were younger (41.5 vs. 45.1 years) and were more likely to perceive they were addicted to smoking cigarettes (87.7% vs. 78.0% perceived being somewhat or very addicted); to report a history of psychiatric/psychological therapy (50.1% vs. 38.2%); to use little cigars, cigarillos, or hookah (41.9% vs. 28.2%); to report having a prior diagnosis of asthma, chronic bronchitis or chronic obstructive pulmonary disease (18.8% vs. 9.7%); and to report participating in zero tobacco-related surveys hosted by GfK during the past year. Interestingly, less than one-third of ENDS users and of non-ENDs users (32.8% and 25.9%, respectively; p = .17) reported ever using an approved nicotine replacement therapy or pharmaceutical drugs to quit smoking. No statistically significant differences were observed at baseline for quit intentions, number of smoking quit attempts in the past year, smoker regret, number of years smoking, cigarettes per day smoked, having strong cravings to smoke, or socio-demographic variables other than age.

**Table 2:** Proportions/Means of covariates measures at baseline by ENDS use^†^.

	Any ENDS Use During Study			No ENDS Use			
	n	%/Mean	95% CI	n	%/Mean	95% CI	*P*
**Perceived Addiction to Smoking Cigarettes**							.033
Not at all	24	5.2	2.7, 9.7	64	16.2	11.6, 22.2	
Yes, somewhat addicted	145	42.6	34.4, 51.1	219	41.7	35.2, 48.6	
Yes, very addicted	164	45.1	36.8, 53.7	208	36.3	30.1, 43.0	
I don’t know	12	7.1	3.2, 15.2	16	5.7	2.8, 11.2	
**Have Strong Cravings to Smoke Cigarettes**							.095
No	71	18.9	13.3, 26.0	133	29.5	23.5, 36.3	
Yes	262	76.7	69.0, 83.0	352	66.8	60.0, 73.1	
Don't know	14	4.4	1.9, 10.1	21	3.7	1.8, 7.4	
**Ever Used Approved NRT or Pharmaceutical Drugs to Quit Smoking**							.17
No	215	67.2	59.0, 74.6	361	74.1	67.8, 79.5	
Yes	132	32.8	25.4, 41.0	145	25.9	20.5, 32.2	
**Use Large Cigars, Little Cigars/Cigarillos, or Hookah**							.011
No	206	58.1	49.4, 66.2	381	71.8	64.9, 77.9	
Yes	141	41.9	33.8, 50.6	123	28.2	22.1, 35.1	
**Marital Status**							.64
Married	154	35.6	28.4, 43.6	242	42.6	36.1, 49.4	
Widowed	8	1.9	0.6, 6.4	25	3.9	2.0, 7.4	
Divorced	51	12.9	7.9, 20.5	70	12.3	8.2, 18.0	
Separated	9	3	1.0, 8.2	9	2	0.8, 5.0	
Never married	65	29.3	21.6, 38.3	90	23.2	17.5, 30.1	
Living with partner	60	17.2	11.8, 24.4	71	16.1	11.8, 21.6	
**Live in a Metropolitan Statistical Area**							.30
Non-Metro	57	16.7	11.3, 24.1	103	21.3	16.2, 27.4	
Metro	290	83.3	75.9, 88.7	404	78.7	72.6, 83.8	
**US Region**							.49
Northeast	65	20.4	14.6, 27.7	85	14.4	10.7, 18.9	
Midwest	104	23	16.5, 31.1	167	25.7	20.3, 31.9	
South	110	33.7	26.1, 42.3	150	37.6	31.0, 44.7	
West	68	22.9	16.5, 30.9	105	22.4	17.1, 28.7	
							
**Children in Household**							.51
No	244	68.5	60.3, 75.7	371	65.1	58.2, 71.3	
Yes	103	31.5	24.3, 39.7	136	34.9	28.7, 41.8	
**Employment Status**							.32
Working—as a paid employee	164	49.9	41.4, 58.3	235	54.1	47.2, 60.8	
Working—self-employed	29	6.1	3.5, 10.4	35	5	2.8, 8.8	
Not working—on temporary layoff from a job	3	0.6	0.2, 2.5	4	2	0.6, 6.6	
Not working—looking for work	28	7.5	4.2, 12.8	38	10	6.4, 15.4	
Not working—retired	40	5.9	3.5, 9.9	98	8	6.0, 10.7	
Not working—disabled	54	20.2	13.4, 29.3	59	12.4	8.5, 17.7	
Not working—other	29	9.8	5.6, 16.6	38	8.5	5.5, 12.8	
**Race/Ethnicity**							.67
White, Non-Hispanic	266	63.1	54.2, 71.2	390	63.7	56.5, 70.3	
Black, Non-Hispanic	26	12.3	7.4, 19.9	43	15.2	10.5, 21.5	
Other, Non-Hispanic	10	6.3	2.7, 14.2	12	4.9	2.5, 9.5	
Hispanic, Any Race	37	17.7	12.0, 25.4	45	14.5	10.0, 20.7	
2+ Races, Non-Hispanic	8	0.5	0.2, 1.4	17	1.7	0.6, 4.6	
**Gender**							.84
Male	178	45.8	37.5, 54.3	267	46.8	40.1, 53.7	
Female	169	54.2	45.7, 62.5	240	53.2	46.3, 59.9	
**Sexual Orientation**							.18
Heterosexual or straight	307	87.5	80.7, 92.1	454	91.4	87.1, 94.4	
Gay	10	2.3	1.0, 5.2	20	1.9	1.0, 3.6	
Lesbian	6	1.8	0.6, 5.2	5	2.3	0.8, 6.3	
Bisexual	14	4.8	2.1, 10.3	17	2.4	1.2, 5.0	
Other	2	0.1	0.0, 0.5	5	0.9	0.2, 3.7	
Refused	8	3.6	1.3, 9.6	6	1	0.3, 3.3	
**Participation in Prior GfK-hosted Tobacco Surveys**							.002
0 surveys	39	19	12.9, 27.3	34	6.6	3.8, 11.1	
1 survey	57	14.6	9.6, 21.6	74	17.9	13.0, 24.1	
2–5 surveys	199	52.5	43.9, 60.9	271	51.7	44.9, 58.5	
6+ surveys	52	13.9	9.0, 20.7	128	23.8	18.6, 29.9	
**Alcohol Consumption (Past Month)**							.20
No	213	60.3	51.6, 68.4	315	67.3	60.5, 73.4	
Yes	133	39.7	31.6, 48.4	188	32.7	26.6, 39.5	
**Ever Received Psychiatric or Psychological Therapy**							.031
No	168	49.9	41.4, 58.5	299	61.8	55.1, 68.2	
Yes	176	50.1	41.5, 58.6	205	38.2	31.8, 44.9	
**Ever Diagnosed with Asthma, Chronic Bronchitis or COPD**							.014
No	294	81.2	72.6, 87.6	440	90.3	86.4, 93.2	
Yes	53	18.8	12.4, 27.4	65	9.7	6.8, 13.6	
**Average Cigarettes** **Smoked per Day**		11.4	10.0, 12.7		10.5	9.4, 11.6	.33
**Years Smoking**		25.2	22.6, 27.9		27.9	25.8, 29.9	.13
**Quit Intentions** **(lower score =** stronger intentions)*		3.97	3.7, 4.2		4.2	4.0, 4.4	.10
**Number of Past Year** **Smoking Quit Attempts**		1.30	1.0, 1.6		0.9	0.7, 1.2	.075
**Smoker Regret** (higher score = more regret)*		1.15	0.9, 1.4		1.06	0.9, 1.2	.47
**Age (years)**		41.5	39.1, 44.0		45.1	43.1, 47.2	.03
Highest Education Received*		9.1	8.7, 9.6		9.4	9.2, 9.7	.22
Household Income*		10.6	9.7, 11.5		10.4	9.8, 11.1	.72
**Perceived Physical Health** **(lower score = better perceived health)**		2.83	2.7, 3.0		2.76	2.7, 2.9	.07

ENDS = electronic nicotine delivery systems. COPD = chronic obstructive pulmonary disease.

^**†**^Sample sizes vary slightly across variables due to varying patterns of data missingness.

*See S1 Table for information regarding the operationalization of these variables.

### Associations between ENDS use and quitting outcomes

While baseline ENDS users did not differ from baseline non-users in their adjusted odds of making a subsequent smoking quit attempt over the next 12 months (53.7% vs. 48.6%; AOR = 0.99, 95% CI = 0.56–1.77) (Table 3, Model 1a), smokers who reported ENDS use at any time during the study period had nearly twice the adjusted odds of making a quit attempt as those who did not use ENDS at all during the same period (58.5% vs. 44.4%; AOR = 1.92, 95% CI = 1.15–3.19) (Model 2a). This latter association was not statistically significant in analysis of multiply imputed data (see S3 Table, Model 2).

**Table 3:** Making a quit attempt and quitting smoking for ≥ 30 days by ENDS use among all baseline smokers (N = 822*) and baseline daily smokers (N = 613*).

		≥ 1 Quit Attempt During Study*						Not Smoking (≥30 days) at Follow-Up				
ENDS Use	Denom	Num	wt. %	95% CI	AOR^†^	95% CI	Denom	Num	wt. %	95% CI	AOR^†^	95% CI
***Model 1a*: *Baseline ENDS Use***												
No ENDS Use at Baseline (Reference)	582	254	48.6	42.20, 55.08	REF	-	582	87	18.9	14.24, 24.68	REF	-
ENDS Use at Baseline	239	120	53.7	43.42, 63.64	0.99	0.56, 1.77	240	25	9.4	5.22, 16.38	0.30	0.13, 0.72
***Model 1b*: *Baseline ENDS Use (Daily Smokers)***												
No ENDS Use at Baseline (Reference)	440	155	38.5	31.25, 46.28	REF	-	440	39	8.03	4.97, 12.73	REF	-
ENDS Use at Baseline	172	73	45.93	33.90, 58.45	1.24	0.65, 2.35	173	13	4.36	1.95, 9.44	0.37	0.13, 1.05
***Model 2a*: *Any ENDS Use***												
No ENDS Use (Reference)	486	197	44.2	37.32, 51.27	REF	-	486	83	22.2	16.70, 28.91	REF	-
Any ENDS Use	335	177	58.5	49.90, 66.66	1.92	1.15, 3.19	336	29	7.7	4.52, 12.92	0.25	0.11, 0.57
ENDS use at baseline & follow-up	123	72	58.8	43.68, 72.46	1.18^a^	0.59, 2.36	124	9	4.8	1.91, 11.44	0.05^a^	0.01, 0.18
ENDS use initiated after baseline	52	31	77.4	59.96, 88.62	5.7^a^	1.95, 16.64	52	3	3.3	0.73, 13.61	0.17	0.02, 1.43
ENDS use but discontinued before follow-up	160	74	50.6	38.48, 62.58	1.83	0.93, 3.58	160	17	12.0	6.01, 22.39	0.70^a^	0.30, 1.62
***Model 2b*: *Any ENDS Use (Daily Smokers)***												
No ENDS Use (Reference)	365	114	31.22	23.99, 39.49	REF	-	365	36	9.17	5.56, 14.77	REF	-
Any ENDS Use	247	114	53.3	43.11, 63.23	2.42	1.36, 4.32	248	16	4.14	1.92, 8.70	0.36	0.12, 1.07
ENDS use at baseline & follow-up	84	43	51.76	33.41, 69.65	1.65^a^	0.76, 3.58	85	6	2.96	0.99, 8.51	0.11^a^	0.03, 0.48
ENDS use initiated after baseline	39	21	76.78	56.44, 89.40	7.49^a^	2.04, 27.50	39	2	1.39	0.28, 6.63	0.22	0.02, 1.91
ENDS use but discontinued before follow-up	124	50	45.63	32.24, 59.68	2.14	1.04, 4.39	124	8	5.92	2.14, 15.31	0.72^a^	0.22, 2.39

ENDS = electronic nicotine delivery systems; Denom = denominator; Num = numerator; wt. = weighted; CI = confidence interval; AOR = adjusted odds ratio; REF = reference.

Statistical adjustments are made for baseline perceptions of addiction, cravings to smoke, cigarettes per day smoked, number of years having smoked, past year quit attempts, use of nicotine replacement theory, poly-use of other combusted tobacco, smoker regret, socio-demographics (age, gender, race/ethnicity, education, household income, MSA status, marital status, sexual orientation, US Census region, children in household), perceived physical health, presence of asthma, chronic bronchitis or COPD, receiving psychological therapy, alcohol consumption, and past year participation in other tobacco studies through GfK.

*Thirty-six cases were dropped from complete-case analyses due to missing data on the covariates, including 8 cases whom reported ENDS use at baseline (cf. Table 1). One additional case was dropped from the analysis for past year quit attempt due to missing data on this outcome variable. See S3 Table for results among all baseline smokers based on multiply imputed data.

^**†**^Exploratory pairwise comparisons were conducted for Models 2 and 2a. AOR with matching superscripts within an outcome model indicate that these groups were significantly different.

The higher likelihood of making at least one quit attempt did not correspond to greater success in quitting smoking (past 30-day abstinence) by follow-up: baseline ENDS users had 70% lower adjusted odds of quitting smoking than baseline non-users (Model 1a: 9.4% vs. 18.9%; AOR = 0.30, 95% CI = 0.13–0.72) and those smokers who reported any ENDS use during the 12-month study had 75% lower odds of quitting smoking than non ENDS-users (Model 2a: 7.7% vs. 22.2%; AOR = 0.25, 95% CI = 0.11–0.57). Those who reported using ENDS throughout the study (i.e., at baseline and follow-up) had the lowest adjusted odds of quitting compared to nonusers (4.8%; AOR = 0.05, 95% CI = 0.01–0.18), as well as those who used ENDS during the study but had discontinued using them before the follow-up survey (*p* < .05). Sensitivity analyses were conducted where we either dropped the minimum abstinence period criterion of 30 days or increased it to six months; the general pattern of results remained consistent.

Turning to analyses of baseline daily smokers, we found no statistically significant association between ENDS use *at baseline* and making at least one subsequent smoking quit attempt (Model 1b), although we did find that those who used ENDS *at any point* during the study period did have 2.4 (95% CI: 1.4, 4.3) times the odds of making a subsequent quit attempt compared to their non-using counterparts (Model 2b). The proportion that reported quitting smoking at follow-up was considerably lower, regardless of ENDS use, for daily smokers compared to the full sample of daily and nondaily smokers. Similar to the full sample, baseline daily smokers who reported using ENDS throughout the study (i.e., at baseline and follow-up) reported significantly lower adjusted odds of quitting smoking at follow-up compared to those who did not use ENDS during the study (3.0% vs. 9.2%; AOR = 0.11, 95% CI = 0.03–0.48) and to those who reported using ENDS during the study but not at follow-up (*p* < .05). Although the adjusted odds of quitting did not differ significantly for other temporal patterns of ENDS use compared to non-use, similar to the overall smoker sample, we observed no instance where ENDS users were more likely to quit than non-ENDS users.

#### ENDS use frequency (Model 3)

Considering the frequency of ENDS use, intentions for using ENDS to quit smoking, use of flavored ENDS, or use of a tank-system ENDS did not substantially change the aforementioned pattern of results (Table 4). Only 19.2% (95% CI = 12.6–28.0) of ENDS users reported any daily use during the study period. Whereas non-daily ENDS users (though not daily users) had higher adjusted odds of making a quit attempt than non-users (59.7% vs. 44.2%; AOR = 2.14, 95% CI = 1.24–3.69), both non-daily (7.5%; AOR = 0.27, 95% CI = 0.11–0.64) and daily (9.3%; AOR = 0.17, 95% CI = 0.04–0.82) ENDS users had lower odds of quitting smoking. In analysis of multiply imputed data, the effects of frequency of ENDS use on making a quit attempt were smaller and statistically non-significant (see S4 Table, Model 3).

**Table 4:** Making a quit attempt and quitting smoking for ≥ 30 days by ENDS use and characteristics of ends use among all baseline smokers (N = 822) and baseline daily smokers (N = 613*).

		Made ≥ 1 Quit Attempt During Study						Not Smoking (≥30 days) at Follow-Up				
ENDS Use During 12-Month Study	Denom	Num	wt. %	95% CI	AOR^†^	95% CI	Denom	Num	wt. %	95% CI	AOR^†^	95% CI
**No ENDS Use (Reference)**	**486**	**197**	**44.2**	**37.32, 51.27**	**REF**	**-**	**486**	**83**	**22.2**	**16.70, 28.91**	**REF**	**-**
**No ENDS Use (Reference–Daily Smokers)**	**365**	**114**	**31.22**	**23.99, 39.49**	**REF**	**-**	**365**	**36**	**9.17**	**5.56, 14.77**	**REF**	**-**
***Model 3a*: *ENDS Use Frequency***												
Non-daily ENDS use	282	146	59.7	50.36, 68.42	2.14	1.24, 3.69	283	19	7.5	4.01, 13.54	0.27	0.11, 0.64
Daily ENDS use	53	31	50.9	30.44, 71.11	0.93	0.32, 2.65	53	10	9.3	4.01, 20.26	0.17	0.04, 0.82
***Model 3b*: *ENDS Use Frequency (Daily Smokers)***												
Non-daily ENDS use	212	94	53.22	42.12, 64.02	2.56	1.37, 4.78	213	7	2.95	0.96, 8.66	0.29	0.08, 1.10
Daily ENDS use	35	20	53.87	30.30, 75.83	1.77	0.48, 6.50	35	9	13.03	5.19, 29.06	0.75	0.18, 3.13
***Model 4a*: *Importance of ENDS Use for Quitting Smoking***												
None or low importance	45	20	44.9	25.23, 66.37	1.35	0.45, 4.06	45	3	8.5	1.49, 36.02	0.16	0.03, 0.87
Moderate to high importance	245	130	60.8	50.85, 70.01	1.8	1.01, 3.21	246	25	8.1	4.48, 14.05	0.24	0.10, 0.59
***Model 4b*: *Importance of ENDS Use for Quitting Smoking (Daily Smokers)***												
None or low importance	29	12	31.74	13.42, 58.25	1.13	0.19, 6.71	29	1	1.19	0.14, 9.30	0.07	0.00, 1.22
Moderate to high importance	181	81	55.46	43.59, 66.73	2.38	1.23, 4.60	182	14	4.02	1.86, 8.49	0.30	0.10, 0.88
***Model 5a*: *ENDS Flavors***												
Tobacco/unflavored	95	42	48.9	32.32, 65.79	1.51	0.57, 3.96	96	3	2.7	0.64, 10.64	0.11	0.02, 0.50
Menthol / Wintergreen / Mint	57	33	60.6	37.76, 79.60	3.41	1.33, 8.71	57	7	6.37	2.22, 16.92	0.38	0.11, 1.36
All other flavors (e.g., fruit, candy)	174	97	60.86	49.52, 71.14	1.83	0.97, 3.48	174	17	8.75	4.43, 16.56	0.22	0.08, 0.59
***Model 5b*: *ENDS Flavors (Daily Smokers)***												
Tobacco/unflavored	78	30	41.59	23.88, 61.77	1.39^a^	0.52, 3.76	79	1	1.05	0.14, 7.49	0.04	0.00, 0.86
Menthol / Wintergreen / Mint	42	25	66.39	37.38, 86.73	6.02^a^	2.28, 15.91	42	5	4.73	1.40, 14.84	0.46	0.10, 2.11
All other flavors (e.g., fruit, candy)	119	55	54.48	40.89, 67.43	2.36	1.13, 4.96	119	8	3.68	1.20, 10.76	0.32	0.09, 1.11
***Model 6a*: *ENDS Device Type***												
Tank ENDS	116	59	53.7	38.86, 67.91	1.53	0.73, 3.19	117	11	11.1	4.85, 23.44	0.39	0.12, 1.27
Cartridge ENDS	149	80	60.76	47.57, 72.55	1.96	1.02, 3.75	149	12	5.45	2.21, 12.84	0.16	0.05, 0.48
Disposable/Other ENDS	70	38	62.06	44.10, 77.23	2.51	0.85, 7.38	70	6	6.4	2.16, 17.46	0.24	0.05, 1.05
***Model 6b*: *ENDS Device Type (Daily Smokers)***												
Tank ENDS	86	39	51.78	34.78, 68.38	2.16	0.89, 5.27	87	6	5.46	1.60, 17.06	0.49	0.11, 2.28
Cartridge ENDS	113	52	50.92	35.97, 65.70	2.26	1.07, 4.78	113	8	2.82	1.15, 6.74	0.19	0.04, 0.87
Disposable/Other	48	23	60.04	37.76, 78.82	3.18	1.06, 9.51	48	2	4.27	0.69, 22.32	0.49	0.08, 3.05

ENDS = electronic nicotine delivery systems; Denom = denominator; Num = numerator; wt. = weighted; CI = confidence interval; AOR = adjusted odds ratio; REF = reference.

Statistical adjustments are made for baseline perceptions of addiction, cravings to smoke, cigarettes per day smoked, number of years having smoked, past year quit attempts, use of nicotine replacement theory, poly-use of other combusted tobacco, smoker regret, socio-demographics (age, gender, race/ethnicity, education, household income, MSA status, marital status, sexual orientation, US Census region, children in household), perceived physical health, presence of asthma, chronic bronchitis or COPD, receiving psychological therapy, alcohol consumption, and past year participation in other tobacco studies through GfK.

*Sample sizes for the models may fluctuate due to missing data on importance of ENDS use for quitting smoking, ENDS flavors, and ENDS device type. See S4 Table for results among all baseline smokers based on multiply imputed data.

^**†**^Exploratory pairwise comparisons among ENDS exposure groups were conducted. AOR with matching superscripts within an outcome model indicate that these groups were significantly different.

Among those who were daily smokers at baseline (Model 3b), 53.8% of daily ENDS users and 53.2% of non-daily ENDS users reported a quit attempt; however, only non-daily ENDS users were significantly more likely to than non-users of ENDS to report a quit attempt (31.2%; AOR = 2.56, 95% CI: 1.2, 3.7). While a greater proportion (13.0%; 95% CI: 5.2%, 29.1%) of baseline daily smokers who used ENDS daily had quit smoking compared to their counterparts who did not use ENDS (9.2%; 95% CI: 5.6%, 14.8%), after adjusting for covariates, the odds of quitting was lower, albeit not statistically significantly lower, for daily ENDS users compared to nonusers.

#### Importance of ENDS use for quitting smoking (Model 4)

Similarly, whereas smokers who indicated that quitting smoking was an important reason for their use of ENDS (the majority) were more likely to report at least one quit attempt (60.8%; AOR = 1.80, 95% CI = 1.01–3.21), odds of quitting smoking were lower for ENDS users regardless of level of importance (important: 8.1%; AOR = 0.24, 95% CI = 0.10–0.59) (Model 4a). Importance of using ENDS for quitting smoking was not associated with making a quit attempt, and the adjusted odds for none or low importance in predicting quitting smoking was not statistically significant in analysis of multiply imputed data (see S4 Table, Model 4).

Similar to the overall smoker sample, daily smokers at baseline who indicated that quitting smoking was an important reason for their use of ENDS were more likely to report at least one quit attempt than their counterparts who did not use ENDS (Model 4b: 55.5% vs. 31.2%; AOR = 2.38, 95% CI = 1.23–4.60). However, they were less likely to have quit smoking a year later (4.0%; AOR = 0.30, 95% CI = 0.10–0.88).

#### ENDS flavors (Model 5)

There was limited evidence that e-liquid flavor might influence quitting rates. Tobacco-flavored or unflavored ENDS users (2.7%; AOR = 0.11, 95% CI = 0.02–0.50) and users of other flavors (e.g., fruit, dessert, spice; 8.8%; AOR = 0.22, 95% CI = 0.08–0.59) had significantly lower adjusted odds of quitting than non-users (Model 5a). In the multiple imputation analysis, only users of other flavors had significantly lower odds of quitting smoking see S4 Table, Model 5). The comparison with non-users’ adjusted odds of quitting was not statistically significant for menthol/wintergreen/mint users. Although the estimated odds of quitting for menthol/mint/wintergreen and other flavor users were more than twice (AOR = 3.4 and 2.0; 95% CI = 0.48–24.1 and 0.38–10.2; respectively) the estimated odds for tobacco/unflavored users, these differences were not statistically significant, possibly due to insufficient statistical power.

Among baseline daily smokers (Model 5b), both menthol/wintergreen/mint users and other flavor users were more likely to report a quit attempt (AORs = 6.0 and 2.4, respectively) than nonusers of ENDS, and menthol/wintergreen/mint users were more likely to report a quit attempt than tobacco/unflavored users (*p* < .05). Only daily smokers who used tobacco/unflavored ENDS were significantly less likely to report quitting smoking (AOR = 0.04) compared to their counterparts who did not use ENDS.

#### ENDS device type (Model 6)

While cartridge ENDS users were significantly more likely to report a subsequent quit attempt (60.1%; AOR = 1.96, 95% CI: 1.02,3.8), they had significantly lower adjusted odds of quitting (5.5%; AOR = 0.16, 95% CI = 0.05–0.48) compared to non-users. The comparisons were similar in direction but not statistically significant for disposable or tank system users (Model 6a). In analysis of multiply imputed data, tank users also had significantly lower rates of quitting (see S4 Table, Model 6). While the odds of quitting for users of tank systems were more than twice the odds of quitting for disposable ENDS users, this difference was nonsignificant (AOR = 2.5, 95% CI = 0.60–10.2).

Among baseline daily smokers, both cartridge and disposable/other ENDS users were more likely to report a subsequent quit attempt (AORs = 2.3 and 3.2, 95% CIs: 1.07, 4.78 and 1.06, 9.51, respectively) compared to their ENDS non-using counterparts. Similar to the full smoker sample, only daily smokers who used cartridge ENDS were significantly less likely to report quitting smoking (AOR = 0.19, 95% CI: 0.04, 0.87).

### ENDS use and smoking intensity among non-quitters

Among participants who were still smoking at follow-up, there were no significant differences observed in the average number of cigarettes per day (CPD) smoked between ENDS users and non-users, regardless of whether we considered ENDS use status only at baseline (Model 7a: Mean CPD = 10.8 vs. 12.2 for baseline ENDS use and non-use, respectively; adj. M_diff_ = -0.56, 95% CI = -1.68–0.56) or at any time during the study (Model 8a: Mean CPD = 11.5 vs. 12.0 for any ENDS use and non-use, respectively; adj. M_diff_ = -0.03, 95% CI = -1.01–0.94) or if analysis is limited to baseline daily smokers (Models 7a and 8a) (Table 5).

**Table 5:** Average daily cigarette consumption at one-year follow-up by ENDS use among non-quitters for all baseline smokers (N = 680*) and baseline daily smokers (N = 543).

		Average Cigarettes per Day Smoked			
ENDS Use	n	wt. Mean	95% CI	Adj. Mean Difference^†^	95% CI
***Model 7a*: *Baseline ENDS Use***					
No ENDS use at Baseline (Reference)	469	12.2	11.03, 13.36	REF	-
ENDS Use at Baseline	211	10.81	9.04, 12.57	-0.56	-1.68, 0.56
***Model 7b*: *Baseline ENDS Use (Daily Smokers)***					
No ENDS use at Baseline (Reference)	386	14.11	12.91, 15.31	REF	-
ENDS Use at Baseline	157	12.76	10.62, 14.91	-0.99	-2.26, 0.29
***Model 8a*: *Any ENDS Use***					
No ENDS Use (Reference)	382	12.02	10.68, 13.35	REF	-
Any ENDS Use	298	11.51	10.06, 12.95	-0.03	-1.01, 0.94
ENDS use at baseline & follow-up	113	8.36	6.53, 10.19	-0.81	-2.27, 0.64
ENDS use initiated after baseline	45	10.28	7.40, 13.15	-0.07	-2.37, 2.23
ENDS use but discontinued before follow-up	140	14.87	12.93, 16.80	0.51	-0.71, 1.74
***Model 8b*: *Any ENDS Use (Daily Smokers)***					
No ENDS Use (Reference)	318	13.94	12.52, 15.36	REF	-
Any ENDS Use	225	13.49	11.91, 15.07	-0.32	-1.42, 0.78
ENDS use at baseline & follow-up	78	9.79	7.36, 12.23	-1.48	-3.19, 0.23
ENDS use initiated after baseline	34	13.02	10.71, 15.33	-0.27	-2.88, 2.34
ENDS use but discontinued before follow-up	113	16.19	14.27, 18.10	0.39	-0.98, 1.76

ENDS = electronic nicotine delivery systems; Denom = denominator; Num = numerator; wt. = weighted; CI = confidence interval; AOR = adjusted odds ratio; REF = reference.

Statistical adjustments are made for baseline perceptions of addiction, cravings to smoke, cigarettes per day smoked, number of years having smoked, past year quit attempts, use of nicotine replacement theory, poly-use of other combusted tobacco, smoker regret, socio-demographics (age, gender, race/ethnicity, education, household income, MSA status, marital status, sexual orientation, US Census region, children in household), perceived physical health, presence of asthma, chronic bronchitis or COPD, receiving psychological therapy, alcohol consumption, and past year participation in other tobacco studies through GfK.

*See S5 Table for results among all baseline smokers based on multiply imputed data.

^**†**^Exploratory pairwise comparisons among ENDS exposure groups were conducted. None of these comparisons was statistically significant.

The lack of clinically meaningful or statistically significant difference in smoking intensity between ENDS users and non-users also held regardless of frequency of ENDS use (Models 9a and 9b), importance of quitting smoking as a reason for using ENDS (Models 10a and 10b), or e-liquid flavor (Models 11a and 11b) for both all smokers and baseline daily smokers (Table 6). In contrast, smokers who reported using disposable/other ENDS reported smoking more cigarettes per day at follow-up than both nonusers of ENDS (adj. M_diff_ = 1.88, 95% CI = 0.15–3.61) and tank system users (*p* < .05) (Model 12a). This pattern also held when analyses were restricted to for baseline daily smokers (Model 12b).

**Table 6:** Average daily cigarette consumption at one-year follow-up by ENDS use and ends use characteristics among non-quitters for all baseline smokers (N = 680*) and baseline daily smokers (N = 543*).

		Average Cigarettes per Day Smoked			
ENDS Use	n	wt. Mean	95% CI	Adj. Mean Difference^†^	95% CI
**No ENDS Use (Reference)**	**382**	**12.02**	**10.68, 13.35**	**REF**	**-**
**No ENDS Use (Reference–Daily Smokers)**	**318**	**13.94**	**12.52, 15.36**	**REF**	**-**
***Model 9a*: *ENDS Use Frequency***					
Non-daily ENDS use	257	11.72	10.19, 13.25	-0.17	-1.19, 0.84
Daily ENDS use	41	10.06	6.03, 14.09	1.14	-1.04, 3.33
***Model 9b*: *ENDS Use Frequency (Daily Smokers)***					
Non-daily ENDS use	201	13.64	12.00, 15.27	-0.37	-1.50, 0.77
Daily ENDS use	24	12.21	6.84, 17.58	0.12	-2.64, 2.87
***Model 10a*: *Importance of ENDS Use for Quitting Smoking***					
None or low importance	39	12.9	8.62, 17.18	1.03	-0.65, 2.72
Moderate to high importance	216	10.27	8.70, 11.83	-0.48	-1.60, 0.65
***Model 10b*: *Importance of ENDS Use for Quitting Smoking (Daily Smokers)***					
None or low importance	25	17.82	14.27, 21.38	0.34	-1.44, 2.12
Moderate to high importance	165	12.08	10.30, 13.87	-0.73	-2.06, 0.60
***Model 11a*: *ENDS Flavors***					
Tobacco/unflavored	91	15.02	12.54, 17.50	0.30	-0.98, 1.57
Menthol / Wintergreen / Mint	50	12.25	8.72, 15.78	0.06	-2.44, 2.56
All other flavors (e.g., fruit, candy)	150	10.32	8.48, 12.16	-0.13	-1.39, 1.13
***Model 11b*: *ENDS Flavors (Daily Smokers)***					
Tobacco/unflavored	77	16.5	13.95, 19.04	-0.14	-1.53, 1.25
Menthol / Wintergreen / Mint	37	14.2	9.97, 18.44	-0.20	-3.01, 2.61
All other flavors (e.g., fruit, candy)	105	12.29	10.25, 14.34	-0.39	-1.83, 1.05
***Model 12a*: *ENDS Device Type***					
Tank ENDS	102	11.85	9.65, 14.05	-0.24^a^	-1.52, 1.03
Cartridge ENDS	135	11.09	8.81, 13.37	-0.92^b^	-2.25, 0.41
Disposable/Other ENDS	61	11.72	8.49, 14.96	1.88^a,b^	0.15, 3.61
***Model 12b*: *ENDS Device Type (Daily Smokers)***					
Tank ENDS	78	13.14	10.83, 15.44	-0.40^a^	-1.82, 1.03
Cartridge ENDS	103	12.81	10.27, 15.34	-1.54^b^	-3.01, -0.07
Disposable/Other ENDS	44	15.33	11.96, 18.70	2.11^a,b^	0.02, 4.20

Statistical adjustments are made for baseline perceptions of addiction, cravings to smoke, cigarettes per day smoked, number of years having smoked, past year quit attempts, use of nicotine replacement theory, poly-use of other combusted tobacco, smoker regret, socio-demographics (age, gender, race/ethnicity, education, household income, MSA status, marital status, sexual orientation, US Census region, children in household), perceived physical health, presence of asthma, chronic bronchitis or COPD, receiving psychological therapy, alcohol consumption, and past year participation in other tobacco studies through GfK.

*Sample sizes for the models may fluctuate due to missing data on importance of ENDS use for quitting smoking, ENDS flavors, and ENDS device type. See S6 Table for results among all baseline smokers based on multiply imputed data.

^**†**^Exploratory pairwise comparisons among ENDS exposure groups were conducted. Adjusted mean difference estimates with matching superscripts within a model indicate that these groups were significantly different.

### Methods used to quit smoking

Table 7 shows the quit methods and resources reported by smokers whom made a quit attempt, either successful or unsuccessful, during the study. Among those who did not use ENDS but had quit smoking, the majority (72.5%) reported quitting by giving up cigarettes all at once (i.e., “cold turkey”). Approximately one-third cut back gradually (35.1%) and relied on the support of friends and family (29.3%). Those who did not use ENDS and were still smoking had similar rates of trying to quit cigarettes all at once (65.7%) as those who had successfully quit, but were more likely to report quitting by gradually cutting back on cigarettes (69.5%). Among those who used ENDS during the study and had quit smoking, a majority reported quitting cigarettes all at once (66.6%), 38.5% reported they had switched completely to ENDS, and 25.7% reported switching partially to ENDS. However, as the sample size for this group is very small (n = 29, of whom 26 reported the method(s) they used to quit smoking), caution is warranted. Among those who used ENDS and were still smoking at the follow-up survey, most reported gradually cutting back on cigarettes in order to quit (71.5%), trying to quit cigarettes all at once (58.7%), and switching partially to ENDS (54.9%).

**Table 7:** Methods used to quit smoking by ENDS use and smoking status at follow-up (N = 374*).

	No ENDS Use (n = 197)								Any ENDS Use (n = 177)							
	Still Smoking (n = 114)				Quit Smoking (n = 83)				Still Smoking (n = 148)				Quit Smoking (n = 29)			
Quit Method or Resource^†^	Denom	Num	wt. %	95% CI	Denom	Num	wt. %	95% CI	Denom	Num	wt. %	95% CI	Denom	Num	wt. %	95% CI
**Cold Turkey**	112	73	65.7	50.1, 78.5	69	56	72.5	54.8, 85.1	147	77	58.7	45.3, 70.9	26	16	66.6	33.3, 88.8
**Gradually Cut Smoking**	112	79	69.5	53.9, 81.6	69	20	35.2	20.8, 52.8	147	109	71.5	57.5, 82.3	26	11	18.7	7.3, 40.0
**Switched Completely to ENDS**	—	—	—	—	—	—	—	—	147	44	29.1	18.7, 42.3	26	12	38.5	15.4, 68.3
**Switched Partially to ENDS**	—	—	—	—	—	—	—	—	147	79	54.9	41.7, 67.4	26	11	25.7	9.5, 53.1
**Nicotine Replacement Therapy**	112	33	29.0	16.9, 45.1	69	5	7.9	2.0, 27.2	146	49	37.8	25.8, 51.6	26	5	11.0	3.4, 28.8
**Cessation Pharmacology**	112	19	12.1	5.5, 24.5	69	5	2.8	1.0, 7.3	147	30	24.3	14.3, 38.3	26	2	1.8	0.32, 9.9
**Counseling, Quit line, Support Group, Internet Resources, Health Professional**	112	10	6.7	3.0, 14.4	69	4	3.8	0.85, 15.2	147	25	17.3	9.3, 29.9	26	1	1.7	0.20, 13.2
**Little Cigars, Filtered Cigars, Cigarillos**	112	6	6.4	1.8, 20.8	69	0	0	—	147	20	17.7	9.5, 30.4	26	1	0.36	0.04, 3.0
**Use Cigars, Snus, Chew, dip/snuff, dissolvables, hookah, Heat-not-Burn**	112	3	2.6	0.68, 9.1	69	2	7.4	1.3, 32.9	147	11	5.2	2.4, 10.9	26	1	0.36	0.04, 3.0
**Support of Friends/Family**	111	31	28.5	17.4, 42.9	68	17	29.3	15.7, 47.9	147	44	31.4	20.7, 44.6	26	6	16.1	5.5, 39.0

ENDS = electronic nicotine delivery systems; Denom = denominator; Num = numerator; wt. = weighted; CI = confidence interval.

*Sample sizes vary by quit method due to missing data.

^†^If the respondent was no longer smoker at the follow-up survey (regardless of duration of abstinence), they were asked to report any of the methods and resources they used “when they quit smoking for good.” If they were still smoking at the follow-up survey, they were asked to report any of the methods or resources they used to “try to quit smoking” since the baseline survey.

## Discussion

The decline of U.S adult smoking rates has accelerated in recent years [75]. In this study, 16% of smokers in 2015 had stopped smoking a year later. However, we found no evidence that ENDS, at least within the context of the US regulatory and tobacco/vaping market landscape during 2015–2016, were helping adult smokers quit at a higher rate than smokers who did not use these products, despite ENDS users being more likely to make a quit attempt. Our findings indicate that, at the time of this study, ENDS under “real world” use and conditions may have suppressed or delayed quitting among some adult smokers, though interpretation of negative effects of ENDS use should consider the high rate of quitting (18%-22%) among non-ENDS users in this study. While this quit rate is higher than a PATH Study estimate for adult smokers, ages 25+ years, who did not use ENDS at wave 1 (11.3%), it is comparable to the quit rate among the PATH younger adult smokers, 18–24 years, 21.3% [76]. Furthermore, among those who had not quit smoking by follow-up, our study did not find evidence that ENDS use was associated with a reduction in cigarette consumption after adjusting for covariates. While aligned with several prior studies [47,71,77–81], these findings diverge from other studies that have found positive associations of ENDS use with quitting smoking [45,61,82,83]. Inconsistencies within the literature have been attributed to the failure of nearly all studies, save the few RCTs, to satisfy six proposed quality standards [53]. Our study may be the only longitudinal cohort study to include the consideration of ENDS “dose,” device type, e-liquid flavors, and whether they are being used for quitting or other purposes. Our results are robust and consistent even after taking into account these factors: regardless of frequency or duration of ENDS use, device type, quitting as reason for use, or e-liquid flavor, ENDS users quit at a lower rate than non-ENDS users. While the few, limited RCT studies indicate the potential of ENDS to help at least some smokers quit, our study, along with a number of population cohort studies, strongly suggest that the potential of ENDS as a disruptive technology capable of helping smokers quit combustibles is not being realized. There are several potential explanations for these findings. First, the effectiveness of ENDS for promoting cessation may be greater for early-adopters (before 2015) [82] compared to later adopters (in 2015–2016), despite the early market dominance of disposable and cig-a-like devices with poorer nicotine delivery. Later adopters of ENDS may differ from early adopters in important yet unidentified ways. Patterns and characteristics of ENDS use may also explain the findings. Many smokers were neither using ENDS daily nor using tank systems despite past research suggesting daily use of advanced systems that offer better nicotine delivery to be predictors of quitting success [61,69,82]. Whereas a recently published analysis of the PATH study found that daily ENDS and tank system users were more likely to have quit smoking cigarettes or reduced their smoking compared to nonusers [84]; another recent, well-designed study of smokers found that few of the smokers that used ENDS post-discharge used them regularly, and that this was associated with lower rates of cessation at 6-months post discharge from the hospital compared to nonusers [85]. Although neither daily use nor use of tank-system ENDS improved quitting over non-ENDS use in this study, this study may have been underpowered to detect higher quitting among tank-system users compared to disposable or cartridge users, and to detect higher quitting among daily users compared to non-daily users. ENDS vary considerably in their features and nicotine delivery across and within subtype and though nicotine delivery among some systems may be comparable to cigarettes, many systems are less efficient in this regard [60,86–91]. Tank systems also may not adequately mimic the experience of smoking a cigarette. Recent innovation and advancements in ENDS engineering, including cartridge-systems (e.g., JUUL), or other nicotine-delivery systems (e.g., heat-not-burn) may offer more appealing and satisfying options to facilitate complete switching for smokers [92,93]. Third, many dual users may use ENDS as a complement, rather than a substitute, to cigarettes [94,95]. In times/places when/where smoking is either prohibited, discouraged or inconvenient, smokers may use ENDS as a way to cope with their cravings in those situations. This type of dual use pattern is unlikely to result in higher quit rates compared to non-ENDS users and is concerning as smoking even one cigarette per day is associated with a substantially higher risk of coronary heart disease and stroke [96]. Lastly, a significant portion of smokers inaccurately believes that ENDS pose higher or similar risks to health as combustible cigarettes [97]. Misinformation and uncertainty about the risks of ENDS relative to smoking may have discouraged complete switching from combustibles to ENDS for many smokers.

Taken together, our results suggest that the current ways that ENDS are used under “real world” conditions may not increase population quit rates and generate meaningful net public health benefits. In the absence of substantial changes in product characteristics that would make ENDS more satisfying and appealing to adult smokers, policies and regulations that incentivize adult smokers to switch to ENDS, and efforts to accurately communicate the risks of ENDS to adult smokers and the general public, a substantial net public health benefit from ENDS in the U.S. seems unlikely. These findings, considered within the context of the current literature, have important regulatory implications. From the perspective of product characteristics, helping smokers quit combustibles will need evidence-based product standards and pre-market reviews that will encourage innovations in products that truly increase population quit rates. For example, the FDA has recently issued an Advance Notice of Proposed Rulemaking (ANPRM) regarding the role of flavors in tobacco product use, including in smokers switching to potentially reduced-harm tobacco products.[98] Much of the research on the impact of flavored ENDS has focused on the toxicity of flavor additives and their appeal to youth, whereas relatively little research has considered their impact on adult smokers. The results of our study suggest whereas the majority of ENDS users reported using flavors other than tobacco or menthol/wintergreen/mint, only the latter were significantly more likely than non-ENDS users to report a subsequent quit attempt, and none of the flavors was associated with greater likelihood of quitting than non-users. In fact, tobacco/unflavored ENDS users, as well as users of fruit, candy, and other flavors (other than menthol or mint) were associated with lower odds of quitting compared to non-ENDS users. Further study of flavors is necessary to better understand how e-liquid flavors influence decisions of smokers to use ENDS and their smoking outcomes.

Moreover, the findings of this and other studies support the notion that focusing on ENDS alone may be insufficient. Regulations and policies that incentivize smokers to switch completely to reduced-harm and reduced-risk products are needed. For example, product standards aimed at reducing the addictiveness of cigarettes may also be required to achieve population harm reduction through switching to lower-harm tobacco products or cessation of all tobacco. This notion is in line with recently announced plans by the FDA to reduce the nicotine within cigarettes while using regulations to promote the availability and acceptability of reduced-harm nicotine and tobacco products (as well as FDA-approved medicinal nicotine products) [99–101]. In addition, education campaigns that accurately communicate the risks of ENDS and other reduced-risk products may also encourage more complete switching from combustibles to ENDS, and in turn boost the potential of ENDS to increase the population quit rate.

## Limitations

Interpretation and drawing of conclusions from this study must be tempered by consideration of its methodological limitations. Foremost, the observational design of this study limits the ability to draw causal inferences. Despite adjustment for an extensive list of potential confounders, we cannot adequately test for unmeasured confounders. Observational studies of nicotine replacement therapy (NRT) have also often failed to find a positive effect on long-term smoking cessation, particularly after NRT became available without a prescription [102,103]. Among other explanations, unmeasured confounders have been cited as a possible reason for these studies to replicate the positive effects observed in RCTs [103]. While additional U.S.-based RCTs that improve upon the weaknesses of past RCTs are much needed, RCTs are limited in their capability of assessing population-level effects of ENDS under real-world use patterns and conditions. Related, alternative approaches for handling observed confounding, such as propensity score weighting or entropy balancing adjustment, might have yielded different results. However, studies examining the association between ENDS use and cessation that have used propensity score weighting or entropy-balancing adjustment have obtained similar results as this study [85,104]. Second, whereas the use of a national probability sample is a strength of this study, use of an online panel prohibited biochemical verification of quitting or ENDS use. While the validity of self-reported cigarette smoking has been supported [105], the accuracy of self-report of ENDS use is less known. Third, although the sample size of this study either exceeds or is comparable to that of similar prior cohort studies, it might have been insufficient for conducting adequately powered comparisons of subgroups of ENDS users. Related, while retention of two-thirds of our sample over the one-year follow-up period is comparable or superior to most similar cohort studies, our reliance on statistical weighting adjustments to address attrition may not fully account for all relevant predictors of missingness. Finally, while this cohort study provides more recent data than other published cohort studies, caution is needed in generalizing its findings to the future given the continued rapid changes in the regulatory and market landscape for tobacco products.

## Conclusions

Our study suggests that use of current ENDS products in real world conditions do not seem to improve the chances of quitting for smokers, and, under the current landscape, may not be the disruptive technology that increases the population quit rate and reduces the harm of combustibles. Additional steps may be needed to spur innovation to create low-harm and low-risk products that adequately deliver nicotine, address the misperceptions of relative harm of ENDS compared to cigarettes, and encourage cessation and complete switching from combustibles to low-harm and low-risk products among smokers who do not want to quit smoking. While this paper advances the current evidence-base by providing more recent data from the first longitudinal cohort study of a moderately large, nationally representative US sample to address recently proposed quality standards, additional research is needed to reconcile the divergent literature and monitor the impact of ENDS in an environment of rapidly evolving markets and regulatory policies.

## Supporting information

S1 Table Detailed description of measures and variable construction. (DOCX)

S2 Table Smoking and ENDS use at one year follow-up for baseline dual users (multiple imputed). (DOCX)

S3 Table Making a quit attempt and quitting smoking for ≥ 30 days by ENDS use (multiple imputed). (DOCX)

S4 Table Making a quit attempt and quitting smoking for ≥ 30 days by ENDS use and characteristics of ends use (multiple imputed). (DOCX)

S5 Table Average daily cigarette consumption at one-year follow-up by ENDS use among non-quitters (multiple imputed). (DOCX)

S6 Table Average daily cigarette consumption at one-year follow-up by ENDS use and ends use characteristics among non-quitters (multiple imputed). (DOCX)

S1 Dataset Raw data file. (CSV)

S1 File Data dictionary for the raw data file. (PDF)
