# Supplementary material for: Are electronic nicotine delivery systems helping cigarette smokers quit? Evidence from a prospective cohort study of U.S. adult smokers, 2015–2016
Source: PLoS One. 2018 Jul 9;13(7):e0198047. doi: 10.1371/journal.pone.0198047 (PMC6037369; doi:10.1371/journal.pone.0198047)
Supplement: S1 Table — (DOCX) [file pone.0198047.s001.docx]

## **Table S1. Detailed Description of Measures and Variable Construction**

| **Construct** | **Question Text [Whom asked / When asked]** | **Response Options** | **Operationalization** |
| --- | --- | --- | --- |
| ***Study Eligibility*** |  |  |  |
| Baseline Smoking Status | (1) Have you smoked at least 100 cigarettes in your entire life? One hundred cigarettes is equal to 5 packs? [Asked: Everyone / Baseline] | 0 = No, 1 = Yes |  |
|  | (2) Do you know smoke cigarettes every day, some days, or not at all) [Asked: If (1) is YES / Baseline] | 0 = Not at all, 1 = Some days, 2 = Everyday | If (1) = Yes and (2) = Some days OR Everyday, then Eligible (Baseline Current Smoker) |
| ***Specific Analysis Eligibility*** |  |  |  |
| Baseline Daily Smoking Status | (1) Have you smoked at least 100 cigarettes in your entire life? One hundred cigarettes is equal to 5 packs? [Asked: Everyone / Baseline] | 0 = No, 1 = Yes |  |
|  | (2) Do you know smoke cigarettes every day, some days, or not at all? | 0 = Not at all, 1 = Some days, 2 = Everyday | If (1) = Yes and (2) = Everyday, then Eligible (Baseline Current Daily Smoker) |
|  |  |  |  |
| ***Primary Outcome*** |  |  |  |
| Not Smoking at Follow-up for > 30 days | (1) Do you know smoke cigarettes every day, some days, or not at all) [Asked: Everyone / Follow-up] | 0 = Not at all, 1 = Some days, 2 = Everyday | 1: (1) = "Not at all" AND (2) = "No" 0: (1) = "Some days" or "Everyday" OR (2) = "Yes" |
|  | (2) In the past 30 days, have you smoked a cigarette, even one or two puffs? [Asked: If (1) is "not at all" or "some days"/ Follow-up] | 0 = No, 1 = Yes |  |
| ***Secondary Outcomes*** |  |  |  |
| Any Past Year Quit Attempt | (1) Do you know smoke cigarettes every day, some days, or not at all) [Asked: Everyone / Follow-up] | 0 = Not at all, 1 = Some days, 2 = Everyday | 1: (1) = "Not at all" OR (2) > 0 0: (1) = "Some days" or "Everyday" AND (2) = 0 |
|  | (2) In total, how many times since August 2015 have you tried to quit smoking cigarettes completely? If you have not tried to quit smoking cigarettes completely since August 2015, please enter 0. [Asked: If (1) is "Everyday" or "Some days"/ Follow-up] | Numeric range: 0-365 |  |
|  |  |  |  |
| Cigarettes Smoked per Day | (1) Do you know smoke cigarettes every day, some days, or not at all) [Asked: Everyone / Follow-up] | 0 = Not at all, 1 = Some days, 2 = Everyday |  |
|  | (2) In the past 30 days, have you smoked a cigarette, even one or two puffs? [Asked: If (1) is "not at all" or "some days"/ Follow-up] | 0 = No, 1 = Yes | IF (1) = Everyday, then =30; else [(3) * (4)] / 30 |
|  | (3) During the past 30 days, on how many days did you smoke cigarettes? [Asked: If (2) is "Yes" / Follow-up] | Numeric range: 1-30 |  |
|  | (4) On average, on the days that you smoke, how many cigarettes a day do you smoke? A pack usually has 20 cigarettes in it. [Asked: If (1) is "Everyday" or "Some days"/ Follow-up] | Numeric range: 1-120 |  |
|  |  |  |  |
| ***Primary Predictor*** |  |  |  |
| Baseline ENDS Use | (1) Have you ever seen or heard of any type of electronic vapor product, such as e-cigarettes, e-cigars, e-hookahs, e-pipes, vape pens, hookah pens or personal vaporizers/mods before this study? [Asked: Everyone / Baseline] | 0 = No, 1 = Yes | 1: (3) = "Rarely", "Some days", or "Every day" 0: (3) = "Not at all" |
|  | (2) Have you ever used electronic vapor products, even one or two times? [Asked: If (1) = "Yes" / Baseline] | 0 = No, 1 = Yes |  |
|  | (3) Do you now use electronic vapor products every day, some days, rarely, or not at all? [Asked: If (2) = "Yes" / Baseline] | 0 = "Not at all", 1 = "Rarely", 2 = "Some days", 3 = "Every day" |  |
|  |  |  |  |
|  |  |  |  |
| Any ENDS Use | (1) Have you ever seen or heard of any type of electronic vapor product, such as e-cigarettes, e-cigars, e-hookahs, e-pipes, vape pens, hookah pens or personal vaporizers/mods before this study? [Asked: Everyone / Baseline] | 0 = No, 1 = Yes |  |
|  | (2) Have you ever used electronic vapor products, even one or two times? [Asked: If (1) = "Yes" / Baseline] | 0 = No, 1 = Yes |  |
|  | (3) Do you now use electronic vapor products every day, some days, rarely, or not at all? [Asked: If (2) = "Yes" / Baseline] | 0 = "Not at all", 1 = "Rarely", 2 = "Some days", 3 = "Every day" |  |
|  | (4) In the past 30 days, have you used electronic vapor products (such as e-cigarettes, e-cigars, e-hookahs, e-pipes, vape pens, hookah pens or personal vaporizers/mods), even one or two times? [Asked: Everyone / Follow-up] | 0 = No, 1 = Yes |  |
|  | (5) Since August 2015, have you used an electronic vapor product, even one or two times? [Asked: If (4) = "No" / Follow-up] | 0 = No, 1 = Yes |  |
|  | (6) Do you now use electronic vapor products every day, some days, rarely, or not at all? [Asked: If (4) = "Yes" OR (5) = "Yes" / Follow-up] | 0 = "Not at all", 1 = "Rarely", 2 = "Some days", 3 = "Every day" |  |
|  |  |  |  |
| *No use at any point* |  |  | (3) = "Not at all" AND (6) = "Not at all" AND (5) = "No" |
| *Continuous use* |  |  | (3) = "Rarely", "Some days", or "Every day" AND(6) = "Rarely", "Some days", or "Every day" AND{ (4) = "Yes" OR (5) = "Yes" } |
| *Use at follow-up and in between, no use at baseline* |  |  | (3) = "Not at all" AND (6) = "Rarely", "Some days", or "Every day" AND { (4) = "Yes" OR (5) = "Yes" } |
| *Use at baseline and/or in between, no use at follow-up* |  |  | (6) = "Not at all" AND { (3) = "Rarely", "Some days", or "Every day" UNION  { (4) = "Yes" OR (5) = "Yes" } } |
|  |  |  |  |
|  |  |  |  |
| ***Frequency of ENDS Use*** | (1) Have you ever seen or heard of any type of electronic vapor product, such as e-cigarettes, e-cigars, e-hookahs, e-pipes, vape pens, hookah pens or personal vaporizers/mods before this study? [Asked: Everyone / Baseline] | 0 = No, 1 = Yes |  |
|  | (2) Have you ever used electronic vapor products, even one or two times? [Asked: If (1) = "Yes" / Baseline] | 0 = No, 1 = Yes |  |
|  | (3) In the past 30 days, have you used electronic vapor products, even one or two times? [Asked: If (2) = "Yes" / Baseline] | 0 = No, 1 = Yes |  |
|  | (4) On how many of the past 30 days have you used electronic vapor products?[Asked: If (3) = "Yes" / Baseline] | Numeric range: 1-30 |  |
|  | (5) Do you now use electronic vapor products every day, some days, rarely, or not at all? [Asked: If (2) = "Yes" / Baseline] | 0 = "Not at all", 1 = "Rarely", 2 = "Some days", 3 = "Every day" |  |
|  | (6) In the past 30 days, have you used electronic vapor products (such as e-cigarettes, e-cigars, e-hookahs, e-pipes, vape pens, hookah pens or personal vaporizers/mods), even one or two times? [Asked: Everyone / Follow-up] | 0 = No, 1 = Yes |  |
|  | (7) During the past 30 days, on how many days did you use electronic vapor products? [Asked: If (6) = "Yes" / Follow-up] | Numeric range: 1-30 |  |
|  | (8) Since August 2015, have you used an electronic vapor product, even one or two times? [Asked: If (6) = "No" / Follow-up] | 0 = No, 1 = Yes |  |
|  | (9) Do you now use electronic vapor products every day, some days, rarely, or not at all? [Asked: If (6) = "Yes" OR (8) = "Yes" / Follow-up] | 0 = "Not at all", 1 = "Rarely", 2 = "Some days", 3 = "Every day" |  |
| *Never daily* |  |  | (4) < 25 AND (5) = "Rarely" or "Some days" AND (7) < 25 AND (9) = "Rarely" or "Some days" |
| *Ever daily* |  |  | (4) >= 25 OR (5) = "Every day" OR (7) >= 25 OR (9) = "Every day" |
|  |  |  |  |
|  |  |  |  |
| ***Importance of ENDS for Quitting Smoking*** | (1) Have you ever seen or heard of any type of electronic vapor product, such as e-cigarettes, e-cigars, e-hookahs, e-pipes, vape pens, hookah pens or personal vaporizers/mods before this study? [Asked: Everyone / Baseline] | 0 = No, 1 = Yes |  |
|  | (2) Have you ever used electronic vapor products, even one or two times? [Asked: If (1) = "Yes" / Baseline] | 0 = No, 1 = Yes |  |
|  | (3) Do you now use electronic vapor products every day, some days, rarely, or not at all? [Asked: If (2) = "Yes" / Baseline] | 0 = "Not at all", 1 = "Rarely", 2 = "Some days", 3 = "Every day" |  |
|  | (4) Electronic vapor products could help me quit smoking regular cigarettes. [Asked: If (3) = "Rarely", "Some days", "Every day" / Baseline] | Numeric range: 0 (Not at all important) - 6 (very important) |  |
|  | (5) DATA ONLY VARIABLE [Follow-up] | 0 = Never EVP user, 1 = Still Current EVP user, 2 = New Current EVP user, 3 = Still Former EVP user, 4 = New Former EVP user |  |
|  | (6) Electronic vapor products could help me quit smoking regular cigarettes. [Asked: If (5) = Still Current EVP user or New Current EVP user / Follow-up] | Numeric range: 0 (Not at all important) - 6 (very important) |  |
| *Non-users* |  |  | Any ENDS use = no use at any point |
| *Low Reason* |  |  | (4) < 3 AND (6) < 3 |
| *High Reason* |  |  | (4) >= 3 OR (6) >= 3 |
| ***E-liquid flavor*** | (1) Have you ever seen or heard of any type of electronic vapor product, such as e-cigarettes, e-cigars, e-hookahs, e-pipes, vape pens, hookah pens or personal vaporizers/mods before this study? [Asked: Everyone / Baseline] | 0 = No, 1 = Yes |  |
|  | (2) Have you ever used electronic vapor products, even one or two times? [Asked: If (1) = "Yes" / Baseline] | 0 = No, 1 = Yes |  |
|  | (3) In the past 30 days, have you used electronic vapor products, even one or two times? [Asked: If (2) = "Yes" / Baseline] | 0 = No, 1 = Yes |  |
|  | (4) Have you ever used electronic vapor products that are: tobacco flavored? [Asked: If (2) = Yes / Baseline] | 0 = No, 1 = Yes |  |
|  | (5) Have you ever used electronic vapor products that are: flavored **but not with tobacco flavor**? [Asked: If (2) = Yes / Baseline] | 0 = No, 1 = Yes |  |
|  | (6) In the past 30 days, have you used electronic vapor products that are flavored (including tobacco flavor)? [Asked: If (3) = YES AND ( (4) = Yes OR (5) = Yes ) / Baseline] | 0 = No, 1 = Yes |  |
|  | (7) Which flavors have you used in electronic vapor products in the past 30 days? Mint, Wintergreen, Menthol [Asked: if (6) = Yes / Baseline] | 0 = No, 1 = Yes |  |
|  | (8) Which flavors have you used in electronic vapor products in the past 30 days? (Fruit e.g. cherry, blueberry, strawberry, watermelon, coconut, etc.) [Asked: if (6) = Yes / Baseline] | 0 = No, 1 = Yes |  |
|  | (9) Which flavors have you used in electronic vapor products in the past 30 days? Coffee (coffee or any related flavor – e.g. espresso, latte, cappuccino, etc.) [Asked: if (6) = Yes / Baseline] | 0 = No, 1 = Yes |  |
|  | (10) Which flavors have you used in electronic vapor products in the past 30 days? Candy or dessert flavors (e.g. caramel, vanilla, chocolate, ice cream, mud pie) [Asked: if (6) = Yes / Baseline] | 0 = No, 1 = Yes |  |
|  | (11) Which flavors have you used in electronic vapor products in the past 30 days? Spice (e.g. clove, cinnamon, nutmeg) [Asked: if (6) = Yes / Baseline] | 0 = No, 1 = Yes |  |
|  | (12) Which flavors have you used in electronic vapor products in the past 30 days? Alcohol or cocktail (e.g. wine, bourbon, rum, brandy, tequila, whiskey, beer, mai-tai, daiquiri) [Asked: if (6) = Yes / Baseline] | 0 = No, 1 = Yes |  |
|  | (13) Which flavors have you used in electronic vapor products in the past 30 days? Tobacco flavor [Asked: if (6) = Yes / Baseline] | 0 = No, 1 = Yes |  |
|  | (14) Which flavors have you used in electronic vapor products in the past 30 days? Some other flavor (specify) [Asked: if (6) = Yes / Baseline] | 0 = No, 1 = Yes |  |
|  | (15) DATA ONLY VARIABLE [Follow-up] | 0 = Never EVP user, 1 = Still Current EVP user, 2 = New Current EVP user, 3 = Still Former EVP user, 4 = New Former EVP user |  |
|  | (16) [IF EVP_2 = CURRENT EVP USER When you now use an electronic vapor product, IF EVP_2 = FORMER EVPUSER When you last used an electronic vapor product], what flavor did you usually use? Mint, Wintergreen, Menthol [Asked: If (15) = Still Current EVP user, or New Current EVP user, or Still Former EVP user, or New Former EVP user / Follow-up] | 0 = No, 1 = Yes |  |
|  | (17) [IF EVP_2 = CURRENT EVP USER When you now use an electronic vapor product, IF EVP_2 = FORMER EVPUSER When you last used an electronic vapor product], what flavor did you usually use? Fruit (e.g. cherry, blueberry, strawberry, watermelon, coconut, etc.) [Asked: If (15) = Still Current EVP user, or New Current EVP user, or Still Former EVP user, or New Former EVP user / Follow-up] | 0 = No, 1 = Yes |  |
|  | (18) [IF EVP_2 = CURRENT EVP USER When you now use an electronic vapor product, IF EVP_2 = FORMER EVPUSER When you last used an electronic vapor product], what flavor did you usually use? Coffee (coffee or any related flavor – e.g. espresso, latte, cappuccino, etc.) [Asked: If (15) = Still Current EVP user, or New Current EVP user, or Still Former EVP user, or New Former EVP user / Follow-up] | 0 = No, 1 = Yes |  |
|  | (19) [IF EVP_2 = CURRENT EVP USER When you now use an electronic vapor product, IF EVP_2 = FORMER EVPUSER When you last used an electronic vapor product], what flavor did you usually use? Candy or dessert flavors (e.g. caramel, vanilla, chocolate, ice cream, mud pie) [Asked: If (15) = Still Current EVP user, or New Current EVP user, or Still Former EVP user, or New Former EVP user / Follow-up] | 0 = No, 1 = Yes |  |
|  | (20) [IF EVP_2 = CURRENT EVP USER When you now use an electronic vapor product, IF EVP_2 = FORMER EVPUSER When you last used an electronic vapor product], what flavor did you usually use? Spice (e.g. clove, cinnamon, nutmeg) [Asked: If (15) = Still Current EVP user, or New Current EVP user, or Still Former EVP user, or New Former EVP user / Follow-up] | 0 = No, 1 = Yes |  |
|  | (21) [IF EVP_2 = CURRENT EVP USER When you now use an electronic vapor product, IF EVP_2 = FORMER EVPUSER When you last used an electronic vapor product], what flavor did you usually use? Alcohol or cocktail (e.g. wine, bourbon, rum, brandy, tequila, whiskey, beer, mai-tai, daiquiri) [Asked: If (15) = Still Current EVP user, or New Current EVP user, or Still Former EVP user, or New Former EVP user / Follow-up] | 0 = No, 1 = Yes |  |
|  | (22) [IF EVP_2 = CURRENT EVP USER When you now use an electronic vapor product, IF EVP_2 = FORMER EVPUSER When you last used an electronic vapor product], what flavor did you usually use? A non-alcoholic drink or beverage (such as soda, energy drinks, or other beverages) [Asked: If (15) = Still Current EVP user, or New Current EVP user, or Still Former EVP user, or New Former EVP user / Follow-up] | 0 = No, 1 = Yes |  |
|  | (23) [IF EVP_2 = CURRENT EVP USER When you now use an electronic vapor product, IF EVP_2 = FORMER EVPUSER When you last used an electronic vapor product], what flavor did you usually use? Tobacco flavor [Asked: If (15) = Still Current EVP user, or New Current EVP user, or Still Former EVP user, or New Former EVP user / Follow-up] | 0 = No, 1 = Yes |  |
|  | (24) [IF EVP_2 = CURRENT EVP USER When you now use an electronic vapor product, IF EVP_2 = FORMER EVPUSER When you last used an electronic vapor product], what flavor did you usually use? Unflavored [Asked: If (15) = Still Current EVP user, or New Current EVP user, or Still Former EVP user, or New Former EVP user / Follow-up] | 0 = No, 1 = Yes |  |
|  | (25) [IF EVP_2 = CURRENT EVP USER When you now use an electronic vapor product, IF EVP_2 = FORMER EVPUSER When you last used an electronic vapor product], what flavor did you usually use? Some other flavor (specify) [Asked: If (15) = Still Current EVP user, or New Current EVP user, or Still Former EVP user, or New Former EVP user / Follow-up] | 0 = No, 1 = Yes |  |
| *Non-user* |  |  | Any ENDS use = no use at any point |
| *Tobacco/unflavored* |  |  | ( (6) = No OR (13) = Yes OR (23) = Yes OR (24) = Yes ) AND do not meet criteria for any category below |
| *Menthol/wintergreen/mint* |  |  | ( (7) = Yes OR (16) = Yes ) AND do not meet criteria for any category below |
| *Other* |  |  | If any of (8) (9) (10) (11) (12) (14) (17) (18) (19) (20) (21) (22) (25) = Yes |
|  |  |  |  |
|  |  |  |  |
| ENDS Device Type | (1) [IF CURRENT EVP USER:Does/ IF FORMER EVPUSER OR DOV_EVP=MISSING: Did] your electronic vapor product use a tank system? | 0 = No, 1 = Yes, 9 = Not sure |  |
|  | (2) [IF CURRENT EVP USER:Does/ IF NEW FORMER EVP USER OR MISSING: Did] your electronic vapor product use a tank system? | 0 = No, 1 = Yes, 9 = Not sure |  |
|  | (3) [IF CURRENT EVP USER): Does/ IF FORMER EVPUSER OR DOV_EVP=MISSING: Did] your electronic vapor product use cartridges? | 0 = No, 1 = Yes, 9 = Not sure |  |
|  | (4) [IF CURRENT EVP USER: Does/ IF NEW FORMER EVP USER OR EVP_2 = MISSING: Did] your electronic vapor product use cartridges? | 0 = No, 1 = Yes, 9 = Not sure |  |
| *Non-user* |  |  | Any ENDS use = no use at any point |
| *Tank* |  |  | (1) = Yes OR (2) = Yes |
| *Cartridge* |  |  | (3) = Yes OR (4) = Yes |
| *Disposable/Other* |  |  | (1) != Yes AND (2) != Yes AND  (3) != Yes AND (4) != Yes |
|  |  |  |  |
|  |  |  |  |
| ***Adjustment Variables*** |  |  |  |
| Age | Gfk Panel Variable | Continuous variable (years) |  |
| Race | Gfk Panel Variable | 1 - White, non-Hispanic; 2 - Black, non-Hispanic; 3 - Other, non-Hispanic; 4 - Hispanic; 5 - 2+ races, non-Hispanic | Dummy-coded with White, non-Hispanic as reference group |
| Gender | Gfk Panel Variable | 1 - Male; 2 - Female |  |
| Income | Gfk Panel Variable | 19 levels: 1 = “Less than $5,000” to 19 = “$175,000 or more” | Unchanged |
| Education | Gfk Panel Variable | 14 levels: 1 = “No formal education” to 14 = “Professional or Doctorate Degree” | Unchanged |
| Marital Status | Gfk Panel Variable | 1 - Married; 2 - Widowed; 3 - Divorced; 4 - Separated; 5 - Never married; 6 - Living with partner | Dummy-coded with Married as reference group |
| Employment Status | Gfk Panel Variable | 1 - Paid employee; 2 - Self-employed; 3 - Temp layoff; 4 - Looking for work; 5 - Retired; 6 - Disabled; 7 - Other | Dummy-coded with Paid employee as reference group |
| MSA Status | Gfk Panel Variable | 0 - Non-Metro; 1 - Metro |  |
| Region | Gfk Panel Variable | 1 - Northeast; 2 - Midwest; 3 - South; 4 - West | Dummy-coded with Northeast as reference group |
| Children in household | Recode of GfK Panel Variable | 4 separate questions about number of children of various ages in household | 0 - No children; 1 - at least 1 child |
| Sexual Orientation | Gfk Panel Variable | 1 - Heterosexual or straight; 2 - Gay; 3 - Lesbian; 4 - Bisexual; 5 - Other | Dummy-coded with Heterosexual as reference group |
| Previous Smoking Studies | # of smoking studies completed since August 2014 | 0 - 0 surveys; 1 - 1 survey; 2 - 2-5 surveys; 3 - 6+ surveys | Dummy-coded with 0 surveys as reference group |
|  |  |  |  |
| Number of years smoking | Gfk Panel variable for Age and "Think about the first time you smoked cigarettes. How old were you at that time?" |  | Computed as current age minus reported age of smoking initiation |
| Nicotine Dependence | "During the past 30 days, on how many days did you smoke cigarettes?" and "On average, on the days that you smoke, how many cigarettes a day do you smoke? A pack usually has 20 cigarettes in it." |  | Computed as (number of days smoked in past 30 days * average daily cigarettes smoked) / 30 |
| Perceived Addiction | "Do you consider yourself addicted to cigarettes?" | 0 - Not at all; 1 - Yes, somewhat addicted; 2 - Yes, very addicted | Unchanged |
| Cravings to Smoke | "Do you ever have strong cravings to smoke cigarettes?" | 0 - No; 1 - Yes | Unchanged |
| Nicotine Replacement | "Have you ever [used nicotine replacements or pharmaceuticals] to try to quit smoking?" | 0 - No; 1 - Yes | Unchanged |
| Traditional Cigars, Little Cigars and Cigarillos, or Hookah use | Do you now [smoke traditional cigars; use little cigars, cigarillos, or filtered cigars; use hookahs] every day, some days, rarely, or not at all? | 0 - Never; 1 - Rarely; 2 - Some days; 3 - every day | 0 - Responded "Never" to all 3 questions; 1 - Used TCs, LCs, or hookah "rarely", "some days", or "every day" |
|  |  |  |  |
| Quit attempts in last 12 months | "In total, how many times in the past 12 months have you tried to quit smoking cigarettes completely?" | 0-365 | Unchanged |
| Quit Intentions | "What best describes your plans regarding quitting smoking?" | 1 - Intent to quit in next 7 days; 2 - Intend to quit in next month; 3 - Intend to quit in next 6 months; 4 - Intend to quit in the next year; 5 - Intend to quit someday, but not within next year; 6 - Never plan to quit | Unchanged |
| Regret Starting Smoking | "If I had it to do over again, I would not have started smoking cigarettes. How much do you agree or disagree?" | -2 - Strongly disagree; -1 - Somewhat disagree; 0 - Neither agree nor disagree; 1 - Somewhat agree; 2 - Strongly agree' | Unchanged |
|  |  |  |  |
| Alcohol Use | "Which of the following have you had to drink in the past month? [alcohol]" | 0 - No; 1 - Yes | Unchanged |
| Counseling/therapy | "Have you ever in your life seen a psychiatrist, psychologist, or social worker for counseling or therapy?" | 1 - Yes; 2 - No | Unchanged |
| Asthma/Bronchitis/COPD | "Have you been diagnosed with [Asthma, chronic bronchitis or COPD]?" | 0 - No; 1 - Yes | Unchanged |
| Perception of health | "In general, would you say your physical health is…" | 1 - Excellent; 2 - Very good; 3 - Good; 4 - Fair; 5 - Poor | Unchanged |
|  |  |  |  |
| ***Quit Methods*** |  |  |  |
|  | [IF (CGNOW_2 = “Some days” OR “Every day” ) AND (CGQTATPY_2 > 0 OR CGQTATPY_2 = -9)] Since August 2015, have you done any of the following to **try to quit smoking**? |  |  |
|  | [IF CGNOW_2 = “Not at all”] Now, think about the time you quit smoking for good. When you quit smoking for good, did you do any of the following? |  |  |
| Cold turkey | Gave up cigarettes all at once? | 0 - No; 1 - Yes |  |
| Gradually cut back | Gradually cut back on cigarettes? | 0 - No; 1 - Yes |  |
| Switched completely to ENDS | Switched **completely** to electronic vapor products such as e-cigarettes, vape-pens, hookah-pens, electronic hookahs (e-hookahs), electronic cigars (e-cigars), electronic pipes (e-pipes), or e-vaporizers? | 0 - No; 1 - Yes |  |
| Switched partially to ENDS | **Substituted some** of my regular cigarettes with electronic vapor products, such as e-cigarettes, vape-pens, hookah-pens, electronic hookahs (ehookahs), electronic cigars (e-cigars), electronic pipes (e-pipes), e-vaporizers, or tanks? | 0 - No; 1 - Yes |  |
| Nicotine Replacement Therapy | Used nicotine replacements like the nicotine patch, nicotine gum, nicotine lozenges, nicotine nasal spray, or nicotine inhaler? | 0 - No; 1 - Yes |  |
| Cessation Pharmacology (non-nicotine) | Used medications like Wellbutrin, Zyban, buproprion, Chantix, or varenicline? | 0 - No; 1 - Yes |  |
| Counseling, etc. | Got counseling, help from a telephone help or quit line, a website such as Smokefree.gov, books, pamphlets, videos, a quit tobacco clinic, class, or support group, or an internet or web-based program, or from a doctor or other health professional? | 0 - No; 1 - Yes |  |
| Little cigars, cigarillos | Used little cigars, filtered cigars or cigarillos to try to quit smoking cigarettes? | 0 - No; 1 - Yes |  |
| Cigars, snus, dip, HnB, etc. | Used any of the following: traditional cigars, snus, chewing tobacco, dip or snuff, dissolvables, hookah, or “heat-not-burn” to **try to quit smoking cigarettes**? | 0 - No; 1 - Yes |  |
| Relied on friends/family | Relied on the support of friends and family to help you quit smoking cigarettes? | 0 - No; 1 - Yes |  |

ENDS = electronic nicotine delivery systems.
