# Supplementary material for: Are electronic nicotine delivery systems helping cigarette smokers quit? Evidence from a prospective cohort study of U.S. adult smokers, 2015–2016
Source: PLoS One. 2018 Jul 9;13(7):e0198047. doi: 10.1371/journal.pone.0198047 (PMC6037369; doi:10.1371/journal.pone.0198047)
Supplement: S2 Table — (DOCX) [file pone.0198047.s002.docx]

## **Table S2. Smoking and ENDS Use at One Year Follow-up for Baseline Dual Users (Multiple Imputed)***

|  | **wt. %** | **95% CI^†^** |
| --- | --- | --- |
| Quit Smoking and Quit ENDS | 6.3 | (2.55, 10.04) |
| Quit Smoking, Using ENDS | 2.63 | (0.34, 4.93) |
| Current Smoker, Quit ENDS | 39.86 | (30.73, 48.98) |
| Dual User (Current Smoker and Using ENDS) | 51.21 | (42.24, 60.18) |

ENDS = electronic nicotine delivery systems. wt. = weighted. CI = confidence interval.

*Estimates and confidence intervals were pooled over 50 imputed datasets, generated from Bayesian Monte Carlo Markov Chain (MCMC) estimation of an unrestricted mean and variance covariance model, using Rubin’s rules.

**^†^**Wald confidence intervals are reported.
