# Supplementary material for: Are electronic nicotine delivery systems helping cigarette smokers quit? Evidence from a prospective cohort study of U.S. adult smokers, 2015–2016
Source: PLoS One. 2018 Jul 9;13(7):e0198047. doi: 10.1371/journal.pone.0198047 (PMC6037369; doi:10.1371/journal.pone.0198047)
Supplement: S3 Table — (DOCX) [file pone.0198047.s003.docx]

## **Table S3. Making a Quit Attempt and Quitting Smoking for ≥ 30 Days by ENDS Use (Multiple Imputed)***

|  |  | **≥ 1 Quit Attempt During Study** | | | | |  |  | **Not Smoking (≥30 days) at Follow-Up** | | | | |  |
| --- | --- | --- | --- | --- | --- | --- | --- | --- | --- | --- | --- | --- | --- | --- |
| **ENDS Use** |  |  | **wt. %** | **95% CI^†^** | **AOR** | **95% CI** |  |  |  | **wt. %** | **95% CI^†^** | **AOR** | **95% CI** |  |
| ***Model 1: Baseline ENDS Use*** |  |  |  |  |  |  |  |  |  |  |  |  |  |  |
| No ENDS Use at Baseline (Reference) |  |  | 56.58 | (51.26, 61.89) | REF | - |  |  |  | 16.68 | (12.26, 21.11) | REF | - |  |
| ENDS Use at Baseline |  |  | 59.37 | (51.54, 67.21) | 0.94 | (0.60, 1.47) |  |  |  | 8.93 | (4.46, 13.4) | 0.4 | (0.21, 0.76) |  |
|  |  |  |  |  |  |  |  |  |  |  |  |  |  |  |
| ***Model 2: Any ENDS Use*** | |  |  |  |  |  |  |  |  |  |  |  |  |  |
| No ENDS Use (Reference) |  |  | 52.85 | (46.64, 59.07) | REF | - |  |  |  | 19.4 | (14.33, 24.48) | REF | - |  |
| Any ENDS Use |  |  | 62.62 | (56.23, 69) | 1.3 | (0.84, 1.99) |  |  |  | 8.59 | (4.03, 13.15) | 0.31 | (0.16, 0.60) |  |
| ENDS use at baseline & follow-up |  |  | 63.16 | (52.74, 73.58) | 1.18 | (0.66, 2.11) |  |  |  | 4.88 | (0.76, 8.99) | 0.14 | (0.05, 0.39) |  |
| ENDS use initiated after baseline |  |  | 64.14 | (48.19, 80.1) | 1.52 | (0.66, 3.49) |  |  |  | 5.02 | (0.00, 12.6) | 0.19 | (0.03, 1.09) |  |
| ENDS use but discontinued before follow-up |  |  | 61.54 | (51.65, 71.42) | 1.3 | (0.74, 2.28) |  |  |  | 12.71 | (4.64, 20.79) | 0.51 | (0.22, 1.15) |  |
|  |  |  |  |  |  |  |  |  |  |  |  |  |  |  |

ENDS = electronic nicotine delivery systems; wt. = weighted; CI = confidence interval; AOR = adjusted odds ratio; REF = reference.

Statistical adjustments are made for baseline perceptions of addiction, cravings to smoke, cigarettes per day smoked, number of years having smoked, past year quit attempts, use of nicotine replacement theory, poly-use of other combusted tobacco, smoker regret, socio-demographics (age, gender, race/ethnicity, education, household income, MSA status, marital status, sexual orientation, US Census region, children in household), perceived physical health, presence of asthma, chronic bronchitis or COPD, receiving psychological therapy, alcohol consumption, and past year participation in other tobacco studies through GfK.

*Estimates and confidence intervals were pooled over 50 imputed datasets, generated from Bayesian Monte Carlo Markov Chain (MCMC) estimation of an unrestricted mean and variance covariance model, using Rubin’s rules.

**^†^**Wald confidence intervals are reported.
