# Supplementary material for: Are electronic nicotine delivery systems helping cigarette smokers quit? Evidence from a prospective cohort study of U.S. adult smokers, 2015–2016
Source: PLoS One. 2018 Jul 9;13(7):e0198047. doi: 10.1371/journal.pone.0198047 (PMC6037369; doi:10.1371/journal.pone.0198047)
Supplement: S4 Table — (DOCX) [file pone.0198047.s004.docx]

## **Table S4. Making a Quit Attempt and Quitting Smoking for ≥ 30 Days by ENDS Use and Characteristics of ENDS Use (Multiple Imputed)***

|  |  | **Made ≥ 1 Quit Attempt During Study** | | | | |  |  | **Not Smoking (≥30 days) at Follow-Up** | | | | | | | | |  |
| --- | --- | --- | --- | --- | --- | --- | --- | --- | --- | --- | --- | --- | --- | --- | --- | --- | --- | --- |
| **ENDS Use During 12-Month Study** |  |  | **wt. %** | **95% CI^†^** | **AOR** | **95% CI** |  |  |  | | **wt. %** | | **95% CI^†^** | | **AOR** | | **95% CI** | |
|  |  |  |  |  |  |  |  |  |  | |  | |  | |  | |  | |
| **No ENDS Use (Reference)** |  |  | **52.85** | **(46.64, 59.07)** | **REF** | **-** |  |  |  | **19.4** | | **(14.33, 24.48)** | | **REF** | | **-** | |  |
|  |  |  |  |  |  |  |  |  |  |  | |  | |  | |  | |  |
| ***Model 3: ENDS Use Frequency*** | |  |  |  |  |  |  |  |  |  | |  | |  | |  | |  |
| Non-daily ENDS use |  |  | 61.77 | (54.55, 69) | 1.28 | (0.80, 2.05) |  |  |  | 8.49 | | (3.52, 13.46) | | 0.32 | | (0.16, 0.65) | |  |
| Daily ENDS use |  |  | 66.82 | (51.5, 82.14) | 1.37 | (0.61, 3.10) |  |  |  | 9.09 | | (0.74, 17.44) | | 0.27 | | (0.08, 0.94) | |  |
| ***Model 4: Importance of ENDS Use for Quitting Smoking*** | | | |  |  |  |  |  |  |  | |  | |  | |  | |  |
| None or low importance |  |  | 55.98 | (36.61, 75.35) | 1.27 | (0.49, 3.31) |  |  |  | 11.73 | | (0.00, 23.96) | | 0.43 | | (0.11, 1.66) | |  |
| Moderate to high importance |  |  | 63.54 | (56.6, 70.49) | 1.3 | (0.82, 2.06) |  |  |  | 8.16 | | (3.42, 12.91) | | 0.3 | | (0.14, 0.60) | |  |
| ***Model 5: ENDS Flavors*** | |  |  |  |  |  |  |  |  |  | |  | |  | |  | |  |
| Tobacco/unflavored |  |  | 58.93 | (45.84, 72.03) | 1.31 | (0.66, 2.60) |  |  |  | 8.91 | | (0.37, 17.45) | | 0.32 | | (0.09, 1.13) | |  |
| Menthol / Wintergreen / Mint |  |  | 58.01 | (40.36, 75.67) | 1.49 | (0.60, 3.69) |  |  |  | 10.14 | | (0.00, 21.21) | | 0.49 | | (0.14, 1.70) | |  |
| All other flavors (e.g., fruit, candy) |  |  | 65.28 | (57.1, 73.45) | 1.24 | (0.73, 2.10) |  |  |  | 8.05 | | (2.8, 13.3) | | 0.27 | | (0.12, 0.61) | |  |
| ***Model 6: ENDS Device Type*** | | |  |  |  |  |  |  |  |  | |  | |  | |  | |  |
| Tank ENDS |  |  | 60.05 | (49.72, 70.38) | 1.11 | (0.62, 1.99) |  |  |  | 8.79 | | (2.85, 14.74) | | 0.31 | | (0.13, 0.73) | |  |
| Cartridge ENDS |  |  | 64.95 | (55.24, 74.67) | 1.48 | (0.85, 2.56) |  |  |  | 8.15 | | (1.64, 14.67) | | 0.33 | | (0.13, 0.85) | |  |
| Other |  |  | 63.33 | (46.1, 80.56) | 1.38 | (0.54, 3.55) |  |  |  | 8.91 | | (-2.08, 19.9) | | 0.25 | | (0.06, 1.16) | |  |
|  |  |  |  |  |  |  |  |  |  |  | |  | |  | |  | |  |

ENDS = electronic nicotine delivery systems; wt. = weighted; CI = confidence interval; AOR = adjusted odds ratio; REF = reference.

Statistical adjustments are made for baseline perceptions of addiction, cravings to smoke, cigarettes per day smoked, number of years having smoked, past year quit attempts, use of nicotine replacement theory, poly-use of other combusted tobacco, smoker regret, socio-demographics (age, gender, race/ethnicity, education, household income, MSA status, marital status, sexual orientation, US Census region, children in household), perceived physical health, presence of asthma, chronic bronchitis or COPD, receiving psychological therapy, alcohol consumption, and past year participation in other tobacco studies through GfK.

*Estimates and confidence intervals were pooled over 50 imputed datasets, generated from Bayesian Monte Carlo Markov Chain (MCMC) estimation of an unrestricted mean and variance covariance model, using Rubin’s rules.

**^†^**Wald confidence intervals are reported.
