# Supplementary material for: Are electronic nicotine delivery systems helping cigarette smokers quit? Evidence from a prospective cohort study of U.S. adult smokers, 2015–2016
Source: PLoS One. 2018 Jul 9;13(7):e0198047. doi: 10.1371/journal.pone.0198047 (PMC6037369; doi:10.1371/journal.pone.0198047)
Supplement: S1 File — (PDF) [file pone.0198047.s008.pdf]

Codebook

CaseID

		Value
Standard Attributes	Label	Case ID
	Measurement	Scale

CGEV100

		Value
Standard Attributes	Label	Have you smoked at least 100 cigarettes in your entire life? One hundred cigarettes is equal to 5 packs.
	Measurement	Nominal
Valid Values	0	No
	1	Yes
Missing Values	-9	Refused

CGNOW

		Value
Standard Attributes	Label	Do you now smoke cigarettes every day, some days, or not at all?
	Measurement	Nominal
Valid Values	0	Not at all
	1	Some days
	2	Everyday
Missing Values	-9	Refused

CGFRQ30

		Value
Standard Attributes	Label	During the past 30 days, on how many days did you smoke cigarettes?
	Measurement	Scale
Labeled Values	-9	Refused

CGDYQTY

		Value
Standard Attributes	Label	On average, on the days that you smoke, how many cigarettes a day do you smoke? A pack usually has 20 cigarettes in it.
	Measurement	Scale
Labeled Values	-9	Refused

CGSTAGE

		Value
Standard Attributes	Label	Think about the first time you smoked cigarettes. How old were you at that time?
	Measurement	Scale
Labeled Values	-9	Refused

CGQTPLN

		Value
Standard Attributes	Label	What best describes your plans regarding quitting smoking cigarettes?
	Measurement	Nominal
Valid Values	1	Intend to quit in the next 7 days
	2	Intend to quit in the next month
	3	Intend to quit in the next 6 months
	4	Intend to quit in the next year
	5	Intend to quit someday, but not within the next year
	6	Never plan to quit
Missing Values	-9	Refused

CGQTATPY

		Value
Standard Attributes	Label	In total, how many times in the past 12 months have you tried to quit smoking cigarettes completely?
	Measurement	Scale
Labeled Values	-9	Refused

CGQSE6

		Value
Standard Attributes	Label	[Used nicotine replacements] Have you ever done any of the following to try to quit smoking?
	Measurement	Nominal
Valid Values	0	No
	1	Yes
Missing Values	-9	Refused

ECFRQ30

		Value
Standard Attributes	Label	On how many of the past 30 days have you used electronic vapor products?
	Measurement	Scale
Labeled Values	-9	Refused

ECNOW

		Value
Standard Attributes	Label	Do you now use electronic vapor products every day, some days, rarely, or not at all?
	Measurement	Nominal
Valid Values	0	Not at all
	1	Rarely
	2	Some days
	3	Every day
Missing Values	-9	Refused

ECTYPRCH

		Value
Standard Attributes	Label	Please think about the electronic vapor product you use/used most of the time. Is/Was your electronic vapor product rechargeable?
	Measurement	Nominal
Valid Values	0	No
	1	Yes
	9	Don't know
Missing Values	-9	Refused

ECTYPCRT

		Value
Standard Attributes	Label	Does/Did your electronic vapor product use cartridges?
	Measurement	Nominal
Valid Values	0	No
	1	Yes
	9	Don't know
Missing Values	-9	Refused

ECTYPTNK

		Value
Standard Attributes	Label	Does/Did your electronic vapor product use a tank system?
	Measurement	Nominal
Valid Values	0	No
	1	Yes
	2	Not sure
Missing Values	-9	Refused

ECFFLV30

		Value
Standard Attributes	Label	In the past 30 days, have you used electronic vapor products that are flavored (including tobacco flavor)?
	Measurement	Nominal
Valid Values	0	No
	1	Yes
Missing Values	-9	Refused

ECFL30_1

		Value
Standard Attributes	Label	[Mint, Wintergreen, Menthol] Which flavors have you used in electronic vapor products in the past 30 days?
	Measurement	Nominal
Valid Values	0	No
	1	Yes
Missing Values	-9	Refused

ECFL30_2

		Value
Standard Attributes	Label	[Fruit (e.g. cherry, blueberry, strawberry, watermelon, coconut, etc.)] Which flavors have you used in electronic vapor products in the past 30 days?
	Measurement	Nominal
Valid Values	0	No
	1	Yes
Missing Values	-9	Refused

ECFL30_3

		Value
Standard Attributes	Label	[Coffee (coffee or any related flavor - e.g. espresso, latte, cappuccino, etc.)] Which flavors have you used in electronic vapor products in the past 30 days?
	Measurement	Nominal
Valid Values	0	No
	1	Yes
Missing Values	-9	Refused

ECFL30_4

		Value
Standard Attributes	Label	[Candy or dessert flavors (e.g. caramel, vanilla, chocolate, ice cream, mud pie)] Which flavors have you used in electronic vapor products in the past 30 days?
	Measurement	Nominal
Valid Values	0	No
	1	Yes
Missing Values	-9	Refused

ECFL30_5

		Value
Standard Attributes	Label	[Spice (e.g. clove, cinnamon, nutmeg)] Which flavors have you used in electronic vapor products in the past 30 days?
	Measurement	Nominal
Valid Values	0	No
	1	Yes
Missing Values	-9	Refused

ECFL30_6

		Value
Standard Attributes	Label	[Alcohol or cocktail (e.g. wine, bourbon, rum, brandy, tequila, whiskey, beer, mai-tai, daiquiri)] Which flavors have you used in electronic vapor products in the past 30 days?
	Measurement	Nominal
Valid Values	0	No
	1	Yes
Missing Values	-9	Refused

ECFL30_7

		Value
Standard Attributes	Label	[Tobacco flavor] Which flavors have you used in electronic vapor products in the past 30 days?
	Measurement	Nominal
Valid Values	0	No
	1	Yes
Missing Values	-9	Refused

ECFL30_9

		Value
Standard Attributes	Label	[Some other flavor] Which flavors have you used in electronic vapor products in the past 30 days?
	Measurement	Nominal
Valid Values	0	No
	1	Yes
Missing Values	-9	Refused

LCNOW

		Value
Standard Attributes	Label	Do you now use little cigars, cigarillos, or filtered cigars every day, some days, rarely, or not at all?
	Measurement	Nominal
Valid Values	0	Not at all
	1	Rarely
	2	Some days
	3	Every day
Missing Values	-9	Refused

TCNOW

		Value
Standard Attributes	Label	Do you now smoke traditional cigars every day, some days, rarely, or not at all?
	Measurement	Nominal
Valid Values	0	Not at all
	1	Rarely
	2	Some days
	3	Every day
Missing Values	-9	Refused

HKNOW

		Value
Standard Attributes	Label	Do you now use hookahs every day, some days, rarely, or not at all?
	Measurement	Nominal
Valid Values	0	Not at all
	1	Rarely
	2	Some days
	3	Every day
Missing Values	-9	Refused

CGCRAVE

		Value
Standard Attributes	Label	Do you ever have strong cravings to smoke cigarettes?
	Measurement	Nominal
Valid Values	0	No
	1	Yes
	9	Don't know
Missing Values	-9	Refused

CGADSF

		Value
Standard Attributes	Label	Do you consider yourself addicted to cigarettes?
	Measurement	Nominal
Valid Values	0	Not at all
	1	Yes, somewhat addicted
	2	Yes, very addicted
	9	I don’t know
Missing Values	-9	Refused

CGREG

		Value
Standard Attributes	Label	If I had it to do over again, I would not have started smoking cigarettes. How much do you agree or disagree with this statement?
	Measurement	Nominal
Valid Values	-2	Strongly disagree
	-1	Somewhat disagree
	0	Neither disagree nor agree
	1	Somewhat agree
	2	Strongly agree
Missing Values	-9	Refused

ECRESN4

		Value
Standard Attributes	Label	[Electronic vapor products could help me quit smoking regular cigarettes] For each reason listed, please indicate how important it is to you in your use of electronic vapor products.
	Measurement	Nominal
Valid Values	0	Not at all important
	1	1
	2	2
	3	3
	4	4
	5	5
	6	Very important
Missing Values	-9	Refused

PPAGE

		Value
Standard Attributes	Label	Age
	Measurement	Scale
Labeled Values	-2	Not asked
	-1	REFUSED

PPEDUC

		Value
Standard Attributes	Label	Education (Highest Degree Received)
	Measurement	Nominal
Valid Values	1	No formal education
	2	1st, 2nd, 3rd, or 4th grade
	3	5th or 6th grade
	4	7th or 8th grade
	5	9th grade
	6	10th grade
	7	11th grade
	8	12th grade NO DIPLOMA
	9	HIGH SCHOOL GRADUATE - high school DIPLOMA or the equivalent (GED)
	10	Some college, no degree
	11	Associate degree
	12	Bachelors degree
	13	Masters degree
	14	Professional or Doctorate degree
Missing Values	-2	Not asked
	-1	REFUSED

PPETHM

		Value
Standard Attributes	Label	Race / Ethnicity
	Measurement	Nominal
Valid Values	1	White, Non-Hispanic
	2	Black, Non-Hispanic
	3	Other, Non-Hispanic
	4	Hispanic
	5	2+ Races, Non-Hispanic
Missing Values	-2	Not asked
	-1	REFUSED

PPGENDER

		Value
Standard Attributes	Label	Gender
	Measurement	Nominal
Valid Values	1	Male
	2	Female
Missing Values	-2	Not asked
	-1	REFUSED

PPINCIMP

		Value
Standard Attributes	Label	Household Income
	Measurement	Scale
Labeled Values	-2	Not asked
	-1	REFUSED
	1	Less than $5,000
	2	$5,000 to $7,499
	3	$7,500 to $9,999
	4	$10,000 to $12,499
	5	$12,500 to $14,999
	6	$15,000 to $19,999
	7	$20,000 to $24,999
	8	$25,000 to $29,999
	9	$30,000 to $34,999
	10	$35,000 to $39,999
	11	$40,000 to $49,999
	12	$50,000 to $59,999
	13	$60,000 to $74,999
	14	$75,000 to $84,999
	15	$85,000 to $99,999
	16	$100,000 to $124,999
	17	$125,000 to $149,999
	18	$150,000 to $174,999
	19	$175,000 or more

PPMARIT

		Value
Standard Attributes	Label	Marital Status
	Measurement	Nominal
Valid Values	1	Married
	2	Widowed
	3	Divorced
	4	Separated
	5	Never married
	6	Living with partner
Missing Values	-2	Not asked
	-1	REFUSED

PPMSACAT

		Value
Standard Attributes	Label	MSA Status
	Measurement	Scale
Labeled Values	-2	Not asked
	-1	REFUSED
	0	Non-Metro
	1	Metro

PPREG4

		Value
Standard Attributes	Label	Region 4 - Based on State of Residence
	Measurement	Nominal
Valid Values	1	Northeast
	2	Midwest
	3	South
	4	West
Missing Values	-2	Not asked
	-1	REFUSED

PPT01

		Value
Standard Attributes	Label	Presence of Household Members - Children 0-1
	Measurement	Scale
Labeled Values	-2	Not asked
	-1	REFUSED

PPT25

		Value
Standard Attributes	Label	Presence of Household Members - Children 2-5
	Measurement	Scale
Labeled Values	-2	Not asked
	-1	REFUSED

PPT612

		Value
Standard Attributes	Label	Presence of Household Members - Children 6-12
	Measurement	Scale
Labeled Values	-2	Not asked
	-1	REFUSED

PPT1317

		Value
Standard Attributes	Label	Presence of Household Members - Children 13-17
	Measurement	Scale
Labeled Values	-2	Not asked
	-1	REFUSED

PPWORK

		Value
Standard Attributes	Label	Current Employment Status
	Measurement	Nominal
Valid Values	1	Working - as a paid employee
	2	Working - self-employed
	3	Not working - on temporary layoff from a job
	4	Not working - looking for work
	5	Not working - retired
	6	Not working - disabled
	7	Not working - other
Missing Values	-2	Not asked
	-1	REFUSED

ppp20063

		Value
Standard Attributes	Label	Q23: Do you consider yourself to be. . .
	Measurement	Nominal
Valid Values	1	Heterosexual or straight
	2	Gay
	3	Lesbian
	4	Bisexual
	5	Other, please specify
Missing Values	-2	Not asked
	-1	Refused

pph10001

		Value
Standard Attributes	Label	Q1: In general, would you say your physical health is. . .?
	Measurement	Nominal
Valid Values	1	Excellent
	2	Very good
	3	Good
	4	Fair
	5	Poor
Missing Values	-2	Not asked
	-1	Refused

pph1brea

		Value
Standard Attributes	Label	Q19: Have you been diagnosed with any of the following medical conditions? [Asthma, chronic bronchitis or COPD]
	Measurement	Nominal
Valid Values	0	No
	1	Yes
Missing Values	-2	Not asked
	-1	Refused

smkstudies

		Value
Standard Attributes	Label	# of smoking studies completed since August 2014
	Measurement	Nominal
Valid Values	0	0 surveys
	1	1 survey
	2	2-5 surveys
	3	6+ surveys

pph10304

		Value
Standard Attributes	Label	Q39: Which of the following have you had to drink in the past month? [None of these]
	Measurement	Nominal
Valid Values	0	No
	1	Yes
Missing Values	-2	Not asked
	-1	Refused

pph10218

		Value
Standard Attributes	Label	Q34: Have you ever in your life seen a psychiatrist, psychologist, or social worker for counseling or therapy?
	Measurement	Nominal
Valid Values	1	Yes
	2	No
Missing Values	-2	Not asked
	-1	Refused

weight

		Value
Standard Attributes	Label	Sample weights: 2016 respondents
	Measurement	Scale

QFLAG_2

		Value
Standard Attributes	Label	DOV: Qualification Flag
	Measurement	Nominal
Valid Values	1	Qualified
	2	Not Qualified
	3	Break-offs
	4	Non-responders

CGNOW_2

		Value
Standard Attributes	Label	Do you now smoke cigarettes every day, some days, or not at all?
	Measurement	Nominal
Valid Values	0	Not at all
	1	Some days
	2	Every day
Missing Values	-9	Refused

CGUSE30_2

		Value
Standard Attributes	Label	In the past 30 days, have you smoked a cigarette, even one or two puffs?
	Measurement	Nominal
Valid Values	0	No
	1	Yes
Missing Values	-9	Refused

CGFRQ30_2

		Value
Standard Attributes	Label	During the past 30 days, on how many days did you smoke cigarettes?
	Measurement	Scale
Labeled Values	-9	Refused

CGDYQTY_2

		Value
Standard Attributes	Label	On average, on the days that you smoke, how many cigarettes a day do you smoke? A pack usually has 20 cigarettes in it.
	Measurement	Scale
Labeled Values	-9	Refused

ECUSE30_2

		Value
Standard Attributes	Label	In the past 30 days, have you used electronic vapor products (such as e-cigarettes, e-cigars, e-hookahs, e-pipes, vape pens, hookah pens or personal vaporizers/mods), even one or two times?
	Measurement	Nominal
Valid Values	0	No
	1	Yes
Missing Values	-9	Refused

ECFRQ30_2

		Value
Standard Attributes	Label	During the past 30 days, on how many days did you use electronic vapor products?
	Measurement	Scale
Labeled Values	-9	Refused

ECUSEYR_2

		Value
Standard Attributes	Label	Since August 2015, have you used an electronic vapor product, even one or two times?
	Measurement	Nominal
Valid Values	0	No
	1	Yes
Missing Values	-9	Refused

ECNOW_2

		Value
Standard Attributes	Label	Do you now use electronic vapor products every day, some days, rarely, or not at all?
	Measurement	Nominal
Valid Values	0	Not at all
	1	Rarely
	2	Some days
	3	Every day
Missing Values	-9	Refused

ECTYPRCH_2

		Value
Standard Attributes	Label	Please think about the electronic vapor product you [use/used] most of the time. [Is/Was] your electronic vapor product rechargeable?
	Measurement	Nominal
Valid Values	0	No
	1	Yes
	9	Don’t know
Missing Values	-9	Refused

ECTYPCRT_2

		Value
Standard Attributes	Label	[Does/Did] your electronic vapor product use cartridges?
	Measurement	Nominal
Valid Values	0	No
	1	Yes
	9	Don’t know
Missing Values	-9	Refused

ECTYPTNK_2

		Value
Standard Attributes	Label	[Does/Did] your electronic vapor product use a tank system?
	Measurement	Nominal
Valid Values	0	No
	1	Yes
	9	Don’t know
Missing Values	-9	Refused

ECFLV_2_1

		Value
Standard Attributes	Label	[Mint, Wintergreen, Menthol] When you now use an electronic vapor product/When you last used an electronic vapor product, what flavor(s) did you usually use?
	Measurement	Nominal
Valid Values	0	No
	1	Yes
Missing Values	-9	Refused

ECFLV_2_2

		Value
Standard Attributes	Label	[Fruit (e.g. cherry, blueberry, strawberry, watermelon, coconut, etc.)] When you now use an electronic vapor product/When you last used an electronic vapor product, what flavor(s) did you usually use?
	Measurement	Nominal
Valid Values	0	No
	1	Yes
Missing Values	-9	Refused

ECFLV_2_3

		Value
Standard Attributes	Label	[Coffee (coffee or any related flavor – e.g. espresso, latte, cappuccino, etc.)] When you now use an electronic vapor product/When you last used an electronic vapor product, what flavor(s) did you usually use?
	Measurement	Nominal
Valid Values	0	No
	1	Yes
Missing Values	-9	Refused

ECFLV_2_4

		Value
Standard Attributes	Label	[Candy or dessert flavors (e.g. caramel, vanilla, chocolate, ice cream, mud pie)] When you now use an electronic vapor product/When you last used an electronic vapor product, what flavor(s) did you usually use?
	Measurement	Nominal
Valid Values	0	No
	1	Yes
Missing Values	-9	Refused

ECFLV_2_5

		Value
Standard Attributes	Label	[Spice (e.g. clove, cinnamon, nutmeg)] When you now use an electronic vapor product/When you last used an electronic vapor product, what flavor(s) did you usually use?
	Measurement	Nominal
Valid Values	0	No
	1	Yes
Missing Values	-9	Refused

ECFLV_2_6

		Value
Standard Attributes	Label	[Alcohol or cocktail (e.g. wine, bourbon, rum, brandy, tequila, whiskey, beer, mai-tai, daiquiri)] When you now use an electronic vapor product/When you last used an electronic vapor product, what flavor(s) did you usually use?
	Measurement	Nominal
Valid Values	0	No
	1	Yes
Missing Values	-9	Refused

ECFLV_2_7

		Value
Standard Attributes	Label	[A non-alcoholic drink or beverage (such as soda, energy drinks, or other beverages)] When you now use an electronic vapor product/When you last used an electronic vapor product, what flavor(s) did you usually use?
	Measurement	Nominal
Valid Values	0	No
	1	Yes
Missing Values	-9	Refused

ECFLV_2_8

		Value
Standard Attributes	Label	[Tobacco flavor] When you now use an electronic vapor product/When you last used an electronic vapor product, what flavor(s) did you usually use?
	Measurement	Nominal
Valid Values	0	No
	1	Yes
Missing Values	-9	Refused

ECFLV_2_9

		Value
Standard Attributes	Label	[Unflavored] When you now use an electronic vapor product/When you last used an electronic vapor product, what flavor(s) did you usually use?
	Measurement	Nominal
Valid Values	0	No
	1	Yes
Missing Values	-9	Refused

ECFLV_2_10

		Value
Standard Attributes	Label	[Some other flavor (specify)] When you now use an electronic vapor product/When you last used an electronic vapor product, what flavor(s) did you usually use?
	Measurement	Nominal
Valid Values	0	No
	1	Yes
Missing Values	-9	Refused

CGQTATPY_2

		Value
Standard Attributes	Label	In total, how many times since August 2015 have you tried to quit smoking cigarettes completely? If you have not tried to quit smoking cigarettes completely since August 2015, please enter 0.
	Measurement	Scale
Labeled Values	-9	Refused

CGQSPY1_2

		Value
Standard Attributes	Label	[Gave up cigarettes all at once?] Since August 2015, have you done any of the following to try to quit smoking?
	Measurement	Nominal
Valid Values	0	No
	1	Yes
Missing Values	-9	Refused

CGQSPY2_2

		Value
Standard Attributes	Label	[Gradually cut back on cigarettes?] Since August 2015, have you done any of the following to try to quit smoking?
	Measurement	Nominal
Valid Values	0	No
	1	Yes
Missing Values	-9	Refused

CGQSPY3_2

		Value
Standard Attributes	Label	[Switched completely to electronic vapor products such as e-cigarettes, vape-pens, hookah-pens, electronic hookahs (e-hookahs), electronic cigars (e-cigars), electronic pipes (e-pipes), or e-vaporizers?] Since August 2015, have you done any of the followin
	Measurement	Nominal
Valid Values	0	No
	1	Yes
Missing Values	-9	Refused

CGQSPY4_2

		Value
Standard Attributes	Label	[Substituted some of my regular cigarettes with electronic vapor products, such as e-cigarettes, vape-pens, hookah-pens, electronic hookahs (e-hookahs), electronic cigars (e-cigars), electronic pipes (e-pipes), e-vaporizers, or tanks?] Since August 2015, h
	Measurement	Nominal
Valid Values	0	No
	1	Yes
Missing Values	-9	Refused

CGQSPY5_2

		Value
Standard Attributes	Label	[Switched to mild or some other brand of cigarettes to try to quit smoking?] Since August 2015, have you done any of the following to try to quit smoking?
	Measurement	Nominal
Valid Values	0	No
	1	Yes
Missing Values	-9	Refused

CGQSPY6a_2

		Value
Standard Attributes	Label	[Used nicotine replacements like the nicotine patch, nicotine gum, nicotine lozenges, nicotine nasal spray, or nicotine inhaler?] Since August 2015, have you done any of the following to try to quit smoking?
	Measurement	Nominal
Valid Values	0	No
	1	Yes
Missing Values	-9	Refused

CGQSPY6b_2

		Value
Standard Attributes	Label	[Used medications like Wellbutrin, Zyban, buproprion, Chantix, or varenicline?] Since August 2015, have you done any of the following to try to quit smoking?
	Measurement	Nominal
Valid Values	0	No
	1	Yes
Missing Values	-9	Refused

CGQSPY7_2

		Value
Standard Attributes	Label	[Got counseling, help from a telephone help or quit line, a website such as Smokefree. gov, books, pamphlets, videos, a quit tobacco clinic, class, or support group, or an internet or web-based program, or from a doctor or other health professional?] Since
	Measurement	Nominal

CGQSPY7_2

		Value
Valid Values	0	No
	1	Yes
Missing Values	-9	Refused

CGQSPY8_2

		Value
Standard Attributes	Label	[Used little cigars, filtered cigars or cigarillos to try to quit smoking cigarettes?] Since August 2015, have you done any of the following to try to quit smoking?
	Measurement	Nominal
Valid Values	0	No
	1	Yes
Missing Values	-9	Refused

CGQSPY9_2

		Value
Standard Attributes	Label	[Used any of the following: traditional cigars, snus, chewing tobacco, dip or snuff, dissolvables, hookah, or “heat-not-burn” to try to quit smoking cigarettes?] Since August 2015, have you done any of the following to try to quit smoking?
	Measurement	Nominal
Valid Values	0	No
	1	Yes
Missing Values	-9	Refused

CGQSPY10_2

		Value
Standard Attributes	Label	[Relied on the support of friends and family to help you quit smoking cigarettes?] Since August 2015, have you done any of the following to try to quit smoking?
	Measurement	Nominal
Valid Values	0	No
	1	Yes
Missing Values	-9	Refused

CGQS1_2

		Value
Standard Attributes	Label	[Gave up cigarettes all at once?] Now, think about the time you quit smoking for good. When you quit smoking for good, did you do any of the following?
	Measurement	Nominal
Valid Values	0	No
	1	Yes
Missing Values	-9	Refused

CGQS2_2

		Value
Standard Attributes	Label	[Gradually cut back on cigarettes?] Now, think about the time you quit smoking for good. When you quit smoking for good, did you do any of the following?
	Measurement	Nominal
Valid Values	0	No
	1	Yes
Missing Values	-9	Refused

CGQS3_2

		Value
Standard Attributes	Label	[Switched completely to electronic vapor products, such as e-cigarettes, vape-pens, hookah-pens, electronic hookahs (e-hookahs), electronic cigars (e-cigars), electronic pipes (e-pipes), e-vaporizers, or tanks?] Now, think about the time you quit smoking f
	Measurement	Nominal
Valid Values	0	No
	1	Yes
Missing Values	-9	Refused

CGQS4_2

		Value
Standard Attributes	Label	[Substituted some of my regular cigarettes with electronic vapor products, such as e-cigarettes, vape-pens, hookah-pens, electronic hookahs (e-hookahs), electronic cigars (e-cigars), electronic pipes (e-pipes), e-vaporizers, or tanks?] Now, think about the
	Measurement	Nominal
Valid Values	0	No
	1	Yes
Missing Values	-9	Refused

CGQS6a_2

		Value
Standard Attributes	Label	[Used nicotine replacements like the nicotine patch, nicotine gum, nicotine lozenges, nicotine nasal spray, or nicotine inhaler?] Now, think about the time you quit smoking for good. When you quit smoking for good, did you do any of the following?
	Measurement	Nominal
Valid Values	0	No
	1	Yes
Missing Values	-9	Refused

CGQS6b_2

		Value
Standard Attributes	Label	[Used medications like Wellbutrin, Zyban, buproprion, Chantix, or varenicline?] Now, think about the time you quit smoking for good. When you quit smoking for good, did you do any of the following?
	Measurement	Nominal
Valid Values	0	No
	1	Yes
Missing Values	-9	Refused

CGQS7_2

		Value
Standard Attributes	Label	[Got counseling, help from a telephone help or quit line, a website such as Smokefree. gov, books, pamphlets, videos, a quit tobacco clinic, class, or support group, or an internet or web-based program, or from a doctor or other health professional?] Now, t

CGQS7_2

		Value
	Measurement	Nominal
Valid Values	0	No
	1	Yes
Missing Values	-9	Refused

CGQS8_2

		Value
Standard Attributes	Label	[Used little cigars, filtered cigars or cigarillos to quit smoking cigarettes?] Now, think about the time you quit smoking for good. When you quit smoking for good, did you do any of the following?
	Measurement	Nominal
Valid Values	0	No
	1	Yes
Missing Values	-9	Refused

CGQS9_2

		Value
Standard Attributes	Label	[Used any of the following: traditional cigars, snus, chewing tobacco, dip or snuff, dissolvables, hookah, or “heat-not-burn” to quit smoking cigarettes?] Now, think about the time you quit smoking for good. When you quit smoking for good, did you do any
	Measurement	Nominal
Valid Values	0	No
	1	Yes
Missing Values	-9	Refused

CGQS10_2

		Value
Standard Attributes	Label	[Relied on the support of friends and family to help you quit smoking cigarettes?] Now, think about the time you quit smoking for good. When you quit smoking for good, did you do any of the following?
	Measurement	Nominal
Valid Values	0	No
	1	Yes
Missing Values	-9	Refused

ECRESN4_2

		Value
Standard Attributes	Label	[Electronic vapor products could help me quit smoking regular cigarettes] The next questions are about the reasons people use electronic vapor products. For each reason listed, please indicate how important it is to you in your use of electronic vapor prod
	Measurement	Nominal

ECRESN4_2

		Value
Valid Values	0	Not at all important
	1	_1
	2	_2
	3	_3
	4	_4
	5	_5
	6	Very important
Missing Values	-9	Refused
